# A Mechanistic Approach to Animal Dispersal—Quantifying Energetics and Maximum Distances

**DOI:** 10.1111/ele.70085

**Published:** 2025-02-20

**Authors:** Caitlin Wilkinson, Ulrich Brose, Alexander Dyer, Myriam R. Hirt, Remo Ryser

**Affiliations:** ^1^ German Centre for Integrative Biodiversity Research Halle‐Jena‐Leipzig Leipzig Germany; ^2^ Institute of Biodiversity, Friedrich‐Schiller‐University Jena Jena Germany; ^3^ Institute for Plant Sciences (IPS), University of Bern Bern Switzerland

**Keywords:** bird, body size, fish, locomotion mode, mammal, traits, transfer

## Abstract

Dispersal is a fundamental process driving many ecological patterns. During transfer, species often make large‐scale displacements resulting in significant energy losses with implications for fitness and survival, however generalising these losses across different taxonomic groups is challenging. We developed a bioenergetic dispersal model based on fundamental processes derived from species traits. By balancing energy storage and energy loss during active dispersal, our mechanistic model can quantify energy expenditures depending on landscape configuration and the species in focus. Moreover, it can be used to predict the maximum dispersal capacity of animals, which we compare with recorded maximum dispersal distances (*n* = 1571). Due to its foundation in bioenergetics it can easily be integrated into various ecological models, such as food‐web and meta‐community models. Furthermore, as dispersal is integral to ecological research, the quantification of dispersal capacities provides valuable insight into landscape connectivity, species persistence, and distribution patterns with implications for conservation research.

## Graphical abstract

Dispersal is a key ecological process, but quantifying the energy costs across species is challenging. We developed a bioenergetic dispersal model that uses species traits to balance energy storage and loss during active dispersal. The model predicts maximum dispersal capacities across taxa and can be integrated into ecological frameworks, offering insights into landscape connectivity, species persistence, and conservation.

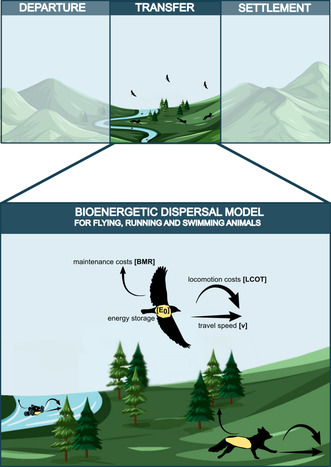

## 1 Introduction

Dispersal is a fundamental process driving biodiversity patterns and is crucial for species survival. Dispersal results in the (re‐)distribution of genes or individuals within and between populations and habitats, affecting local adaptations, extinction risks, population connectivity, metapopulation dynamics, and the overall distribution of species (Kokko and Lopez‐Sepulcre 2006; Matthysen 2012). Events such as climate change (e.g., temperature fluctuations), biological invasions, and habitat fragmentation can severely alter dispersal ecology and evolution (Travis et al. 2013). Thus, predicting an animals' dispersal capacity can help us understand ecological patterns and dynamics as well as a species' ability to cope with global changes.

Dispersal is complex with a myriad of definitions and interacting drivers which affect dispersal success (Matthysen 2012). In general, dispersal can be divided into three distinct phases (departure, transfer and settlement) with substantial costs and varying drivers at every phase (Bonte et al. 2012; Bowler and Benton 2005). In line with the movement paradigm (Nathan et al. 2008), the departure phase of dispersal is primarily influenced by an organism's internal state—specifically, its life stage driving the need to disperse (Bonte et al. 2012). In contrast, the transfer phase is driven by its locomotion capacity—how far and fast the animal can move (Spiegel et al. 2017). Regarding navigation capacity, dispersal is characterised by a strong drive to move away in response to population density or suboptimal conditions rather than toward a specific destination (Bowler and Benton 2005). This contrasts with migration, which involves cyclical tracking of resources or mates often guided by genetically fixed routes (Jeltsch et al. 2013).

The costs of dispersal have been classified into energy costs of movement, time costs by investing in dispersal rather than other activities, risks endured during movement with increased predation or settlement in unsuitable habitats and opportunity costs including the loss of previous advantages at the origin site (Bonte et al. 2012). The most energetically costly phase of active dispersal is transfer, where animals often make large‐scale displacements, resulting in significant energy losses that are typically assumed and not directly tested (Benoit et al. 2020; Klarevas‐Irby et al. 2021). These energetic demands are important for understanding and predicting dispersal capacity with likely implications for the settlement phase. Despite technological advancements in the field of tracking devices (Wilmers et al. 2015), it is still hard to empirically quantify the energetics of dispersal with evidence only in a few species (Benoit et al. 2020; Klarevas‐Irby et al. 2021), potentially due to the costs and impracticality of such methods at large spatial scales or across taxonomic groups. One alternative approach to estimate energetic costs of dispersal is to measure body condition before and after the transient phase. Body condition has been used as a proxy for a species' energy reserves in several studies of dispersal (e.g., Peig and Green 2009; Robles et al. 2022). However, the non‐directional nature of dispersal makes it difficult to measure body condition before and after transfer, hindering a systematic assessment of energy costs. Alternatively, mechanistic models can provide a crucial tool linking species traits to long‐distance dispersal (e.g., Nathan et al. 2003), offering a potential solution to address this gap. For instance, energy costs of dispersal can be approximated by using fundamental biological processes such as basal metabolic demands and locomotion costs, which can be predicted from species traits—similar to mechanistic models of migration (e.g., Hein et al. 2012). Another advantage of mechanistically quantifying the energetics of dispersal is that it allows estimation of maximum dispersal distances, which can be relevant for assessing habitat isolation or landscape connectivity (Jenkins et al. 2007). Furthermore, such models enable integration with mechanistic models capturing the departure phase, which is linked to population growth or dynamics (e.g., Kot et al. 1996), as well as the settlement phase to inform immigration rates (e.g., Whittaker et al. 2008).

In this study, we present a mechanistic bioenergetic dispersal model quantifying the dispersal costs during the transient phase based on energy storage (i.e., fat reserves) and energy loss (i.e., maintenance and locomotion costs), which are dependent on an animal's body mass, taxonomic group (i.e., bird, mammal, fish), and locomotion mode (i.e., flying, running, swimming). The model enables quantification of energetic costs for any realised dispersal distance and prediction of the maximum distance animals can disperse in a straight line.

## 2 Method

### 2.1 Bioenergetic Dispersal Model

**FIGURE 1:**
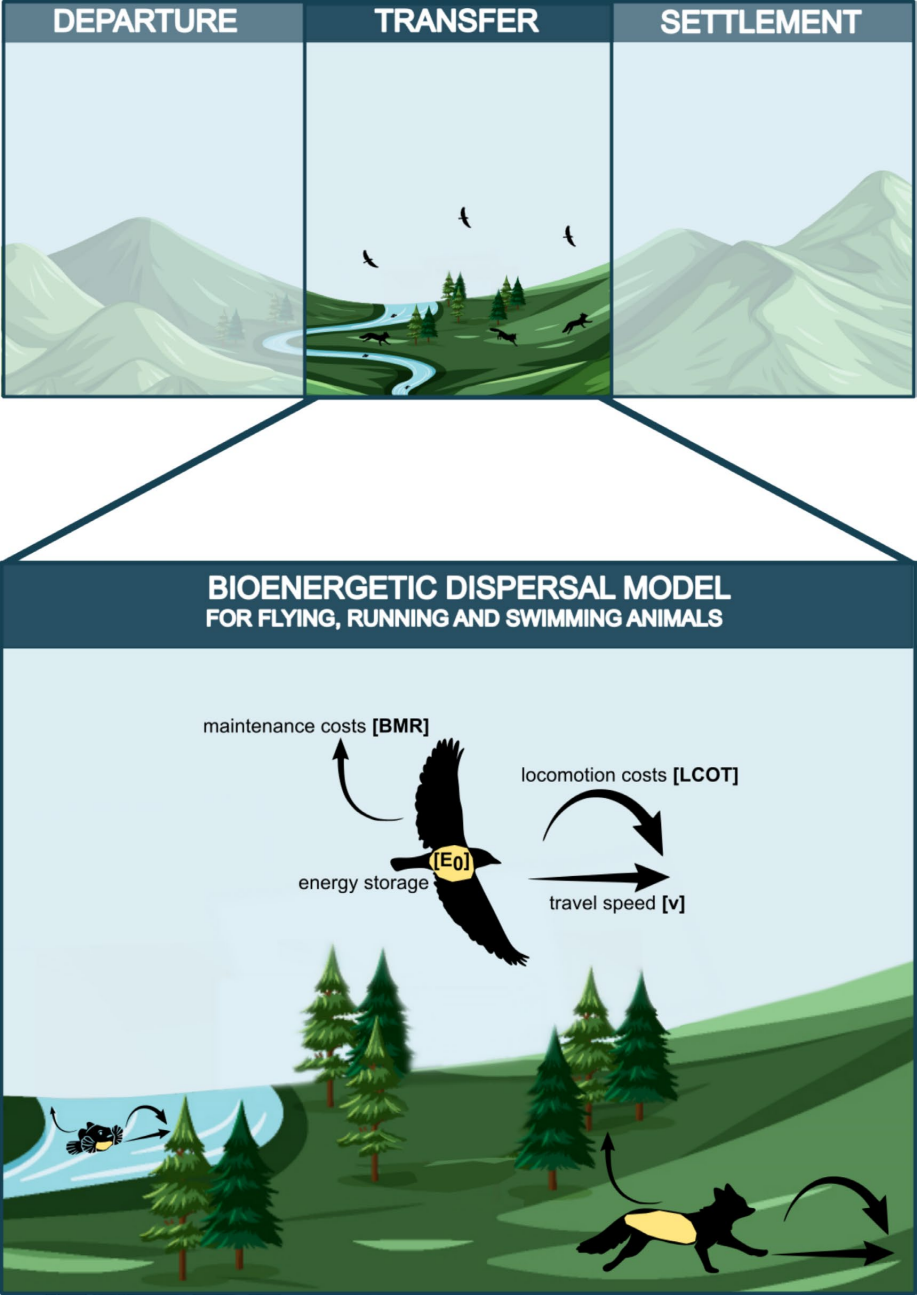
Conceptual illustration of the bioenergetic dispersal model for flying, running and swimming animals during the transfer phase of active dispersal. The model incorporates energy storage (i.e., the fat reserves an animal has) represented by $E_{0}$ and energy costs. The energetic costs during dispersal are a combination of an animals' maintenance costs (i.e., basal metabolic rate, BMR) and the locomotion costs per unit time (LCOT) as a function of travel speed v.

In the context of this study, we classify dispersal as the active movement of species during the transfer phase, over distances greater than an animals' home and exploratory ranges. We developed a bioenergetic dispersal model based on energy storage (i.e., fat reserves) and costs. This model applies similar concepts to dispersal as previous models of animal migration (e.g., Hein et al. 2012), accounting for both the energetic costs of maintenance (here approximated by the basal metabolic rate) and locomotion (here costs of transport and travel speed) of animals (Figure 1). We parameterized the model for flying birds, running mammals, and swimming fishes based on data availability.

#### 2.1.1 Energetics of Dispersal

The remaining energy storage $E_{r}$ at any given distance or time can be calculated by subtracting the energetic costs $E_{c}$ from the initial energy storage $E_{0}$:

(1) $$E_{r}=E_{0}-E_{c}$$

To quantify the energetic costs of dispersal $E_{c}$, we calculate the energetic demands over time $t_{1}$:

(2) $$E_{c}={\mathrm{FMR}}_{\mathrm{dis}}\cdot t_{1}$$

The energy demand during dispersal is defined as the field metabolic rate ${\mathrm{FMR}}_{\mathrm{dis}}$, which is the sum of an animals' maintenance costs (i.e., basal metabolic rate [BMR]) and locomotion costs (LCOT) per unit time, where locomotion costs are a function of travel speed v (Peters 1986):

(3) $${\mathrm{FMR}}_{\mathrm{dis}}=\mathrm{BMR}+f_{\text{LCOT}}\left(v\right)$$

In both flying and running, an additional parameter is included to account for the postural costs that the animal experiences (Peters 1986) (Table 1, parameter $a_{13}$ – $\;a_{14}$). Further, the time $t_{1}$ an animal moves depends on the total distance travelled *D* and its travel speed v:

(4) $$t_{1}=\frac{D}{v}$$

**TABLE 1:** Overview of parameters used for calculating energy storage, basal metabolic rate, travel speed, locomotion costs, and field metabolic rate across different groups (taxonomic groups or locomotion modes). The table includes coefficients (at mass = 1 kg) and slopes, with values converted from the original references. Detailed information on unit conversions is available in Table S1 of the Supporting Information.

Output		Equation	Group	Parameter		Value	Units	Reference
Energy storage [*J*]	$E_{0}$	$a\;M^{b}$	Birds	Coefficient	$a_{1}$	$2.5\times 10^{6}$	$J\;{\mathrm{kg}}^{-b_{1}}$	Antoł and Kozłowski (2020)
				Exponent	$b_{1}$	0.98	NA	
			Mammals	Coefficient	$a_{2}$	$2.00\times 10^{6}$	$J\;{\mathrm{kg}}^{-b_{2}}$	
				Exponent	$b_{2}$	1.00	NA	
			Fish	Coefficient	$a_{3}$	$4.78\times 10^{6}$	$J\;{\mathrm{kg}}^{-b_{3}}$	Martin et al. (2017)
				Exponent	$b_{3}$	1.02	NA	
Basal metabolic rate [$J\;s^{-1}$]	BMR	$a\;M^{b}$	Birds	Coefficient	$a_{4}$	3.63	$J\;s^{-1}{\mathrm{kg}}^{-b_{4}}$	Gavrilov et al. (2022)
				Exponent	$b_{4}$	0.65	NA	
			Mammals	Coefficient	$a_{5}$	2.89	$J\;s^{-1}{\mathrm{kg}}^{-b_{5}}$	
				Exponent	$b_{5}$	0.74	NA	
			Fish	Coefficient	$a_{6}$	0.29	$J\;s^{-1}{\mathrm{kg}}^{-b_{6}}$	Watanabe and Payne (2023)
				Exponent	$b_{6}$	0.95	NA	
Travel speed [$m\;s^{-1}$]	v	$\frac{\left(\frac{1}{k}\right)M^{c}}{M^{\left(c+d_{1}\right)}+\left(\frac{1}{k\cdot v_{0}}\right)}$	Flying	Coefficient	$v_{0\mathrm{fly}}$	30.54	$m\;s^{-1}\;{\mathrm{kg}}^{-c}$	Dyer et al. (2023)
			Running	Coefficient	$v_{0\mathrm{run}}$	0.28	$m\;s^{-1}\;{\mathrm{kg}}^{-c}$	
			Swimming	Coefficient	$v_{0\text{swim}}$	0.39	$m\;s^{-1}\;{\mathrm{kg}}^{-c}$	
			All	Exponent	c	0.27	NA	
				Coefficient	k	0.03	$s\;m^{-1}\;{\mathrm{kg}}^{-d}$	
				Exponent	d	0.24	NA	
Locomotion costs [$J\;s^{-1}$]	LCOT	$\;a_{7}M^{b_{7}}\cdot v^{b_{8}}+a_{8}M^{b_{7}}\cdot v^{b_{9}}+a_{9}M^{b_{10}}\cdot v^{b_{9}}$	Flying	Coefficient	$a_{7}$	32.00	$J\;s^{-1}$	Tucker (1973) in Peters (1986) (Chapter 6)
				Coefficient	$a_{8}$	$3.30\times 10^{-3}$		
				Coefficient	$a_{9}$	$5.80\times 10^{-3}$		
				Exponent	$b_{7}$	0.66	NA	
				Exponent	$b_{8}$	−1.00	NA	
				Exponent	$b_{9}$	2.50	NA	
				Exponent	$b_{10}$	0.49	NA	
		$a_{10}M^{b_{11}}\cdot v$	Running	Coefficient	$a_{10}$	11.30	$J\;s^{-1}$	Peters (1986) (Chapter 6)
				Exponent	$b_{11}$	0.72	NA	
		$a_{11}\exp\cdot \left(\;a_{12}M^{b_{13}}\cdot v\right)$	Swimming	Coefficient	$a_{11}$	0.12	$J\;s^{-1}$	Beamish (1978) in Peters (1986) (Chapter 6)
				Coefficient	$a_{12}$	1.88		
				Exponent	$b_{12}$	−0.36	NA	
Field metabolic rate [$J\;s^{-1}$]	${\mathrm{FMR}}_{\mathrm{dis}}$	$a_{13}\mathrm{BMR}+\text{LCOT}$	Flying	Coefficient	$a_{13}$	1.10	$J\;s^{-1}$	Peters (1986) (Chapter 6)
		$a_{14}\mathrm{BMR}+\text{LCOT}$	Running	Coefficient	$a_{14}$	1.20	$J\;s^{-1}$	
		BMR+LCOT	Swimming				$Js^{-1}$	

We derive both energy storage and energetic cost parameters from species traits including body mass, locomotion mode, and taxonomic group (see below).

#### 2.1.2 Energy Storage

**FIGURE 2:**
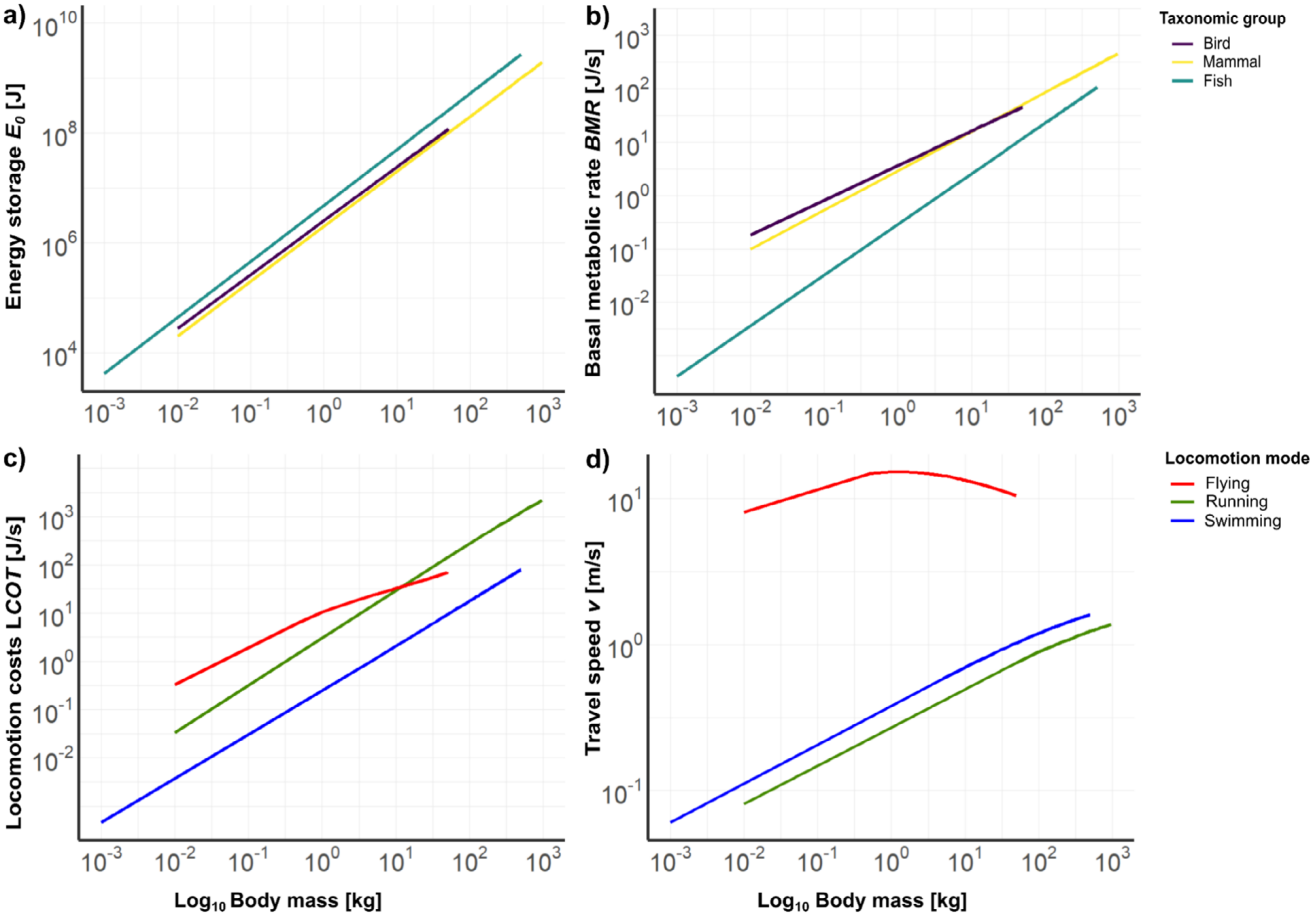
Allometric relationships of the processes underlying the bioenergetic dispersal model: (a) energy storage $E_{0}$; (b) basal metabolic rate BMR; (c) locomotion costs (per time) LCOT; (d) travel speed v. The colours represent either taxonomic group (bird, mammal, fish) or locomotion mode (flying, running, swimming).

For long‐distance movement, like dispersal, an animal relies on its energy storage $E_{0}$ to sustain movement for longer periods and distances. As the mass‐scaling of fat varies across different taxonomic groups (Figure 2a), we applied taxon‐specific relationships in the model (Table 1, parameter $a_{1}$ – $\;b_{3}$).

#### 2.1.3 Energy Costs

The energy demands during dispersal $\left({\mathrm{FMR}}_{\mathrm{dis}}\right)$ comprise the basal metabolic rate *BMR* and the locomotion costs *LCOT* (Equation 3), which is the energy animals need to move as a function of their body mass *M* and travel speed v. Here, we also applied taxon‐specific scaling relationships for BMR (Table 1, parameter $a_{4}$ – $\;b_{6}$; Figure 2b) and additionally locomotion‐specific functions of LCOT (Peters 1986) (Table 1; parameter $a_{7}$ – $\;b_{13}$; Figure 2c). The travel speed v of dispersing animals is predicted from a general locomotion model that jointly accounts for body‐mass dependent constraints on an animals' energy utilisation and heat‐dissipation capacities (Dyer et al. 2023). Thereby, balancing the trade‐off between more energy cost‐efficient dispersal at high speeds and the increased risk of hyperthermia arising from the build‐up of metabolic heat (Dyer et al. 2023). The travel speed varies depending on locomotion mode, which was accounted for in the model (Table 1, parameter $v_{0\mathrm{fly}}$ – d; Figure 2d).

#### 2.1.4 Maximum Dispersal Distance

This bioenergetic dispersal model also allows for the quantification of the physiological maximum dispersal distance *D*_max_ of an animal:

(5) $$D_{\max}=v\cdot t_{2}$$

v is the animals' travel speed and $\;t_{2}$ is the total time it can travel until its available energy is depleted. We assume residual energy is needed upon arrival for an animal to survive and fulfil its daily tasks. We define this residual energy threshold as a fraction λ of the energy storage $E_{0}$, thus the available energy for dispersal is: $E_{\alpha }=\left(1-\lambda \;\right)\cdot E_{0}$. For our analyses, we use λ equal to 0.1, meaning 10% of the total energy storage is needed upon arrival. Due to a lack of empirical evidence on the value of 𝝀, we performed additional sensitivity analyses across a range of 𝝀 values (see Figure S1). To determine time $t_{2}$ at which energy has been depleted, we divide the available energy by the field metabolic rate ${\mathrm{FMR}}_{\mathrm{dis}}$:

(6) $$t_{2}=\frac{E_{\alpha }}{{\mathrm{FMR}}_{\mathrm{dis}}}$$

Finally, substituting $t_{2}$ in Equation (4) with Equation (6) results in the physiological maximum possible dispersal distance of a given animal based on locomotion mode and related taxonomic group.

#### 2.1.5 Adding Resting Time or Stop‐Overs

During dispersal, animals might stop to rest, hide to avoid predation or forage for food. Resting times can be easily included into the model by splitting the time budget into resting and moving times. Considering β as the time spent resting, this would yield:

$$D_{\max\left(\beta \right)}=\left(1-\beta \right)\cdot v\cdot t_{3}$$

$$t_{3}=\frac{E_{\alpha }}{\left(1-\beta \right)\cdot {\mathrm{FMR}}_{\mathrm{dis}}+\beta \cdot \mathrm{BMR}}$$

Similarly, time spent foraging could be included by adding energy gain through feeding, which can be expressed by a functional response (Holling 1959; Wootton et al. 2023), which can take various mechanisms of the predator–prey interaction into account such as prey density, movement speed, capture success, and handling time.

#### 2.1.6 Predicting Dispersal‐Cost‐Weighted Spatial Networks

The predicted physiological maximum dispersal distances can be used to model landscape connectivity across different body sizes and locomotion modes. In this context, a link is present in the spatial network only when a new patch is present within the animal's maximum dispersal distance. Furthermore, the bioenergetic model allows for the calculation of energetic costs associated with moving between landscape patches, creating what we term a ‘dispersal‐cost‐weighted spatial network’. To calculate these costs, the time $t_{1}$ required to travel between patches is first derived using Equation (4) and using known distances between patches. This travel time $t_{1}$ is then used in Equation (2) to calculate the total energy costs $E_{c}$ of the journey. From this, we can derive a dispersal‐cost‐weighted spatial network by determining the relative energy remaining after travel, expressed as $1-\frac{E_{c}}{E_{\alpha }}$ (i.e., the energy costs relative to the animals' available energy for dispersal $E_{\alpha }$).

We demonstrate this for a small (4.5 kg) and a large (4000 kg) running mammal species. We created two exemplary landscapes, one with a random and one with a clustered (e.g., spatially autocorrelated) patch‐location distribution. By assigning a cartesian scale to that landscape, we calculated Euclidean distances between all patches. For each species and each landscape, a dispersal‐cost‐weighted spatial network was then calculated. We applied the bioenergetic dispersal model to estimate the energetic costs associated with each distance. Negative energy values (indicating energetic costs exceeding the energy available) were set to zero to indicate dispersal links that are not possible for each species. To assess dispersal potential and landscape connectivity, we calculated the proportion of possible dispersal links within the network, that is, the connectance (based on presence‐absence of dispersal links) and the weighted connectance (based on cost‐weighted dispersal links) for each species and landscape type.

### 2.2 Empirical Data

#### 2.2.1 Dispersal Dataset

To compare and discuss the model predictions for maximum dispersal distance with recorded maximum dispersal distances, we obtained empirical data from peer‐reviewed published studies using the Web of Science Core Collection. Due to the taxonomic breadth of our search, we used search terms to identify metastudies of dispersal distances or studies focusing on multiple species and taxa. The combinations of the following terms were used: (“dispersal distance” OR “dispersal distance data” OR “dispersal distances”) NOT (“single species” OR “individual species” OR “one species” OR “case study”) AND (organism* OR animal* OR fauna* OR vertebrate* OR mammal* OR bird* OR avian*) AND (terrestrial OR marine OR aquatic OR freshwater). These search terms rendered 212 papers. Additionally, we included suitable papers from the reference list of papers found through the literature search. We included data from field and laboratory studies that reported dispersal distances of individuals or groups of species that actively dispersed. We included only maximum dispersal distance data and excluded mean or median dispersal distances, values calculated using genetic methods, any passive or wind dispersal distance (i.e., terms such as propagules, particles, dust, seed, rafting, larval), any simulated dispersal distances, and any non‐distinct values (i.e., using ranges, words and symbols) (*n* = 2152 of 4427). To add trait data, we first standardised the scientific names of species, which were extracted from our raw datafile and all trait resources we used. The scientific names of species were then defined by the R package *rgbif* (Chamberlain et al. 2024), where we checked the extracted binomial names of species and corrected any spelling mistakes or outdated classifications using the GBIF database. To fill in body masses, we used secondary trait resources including Animal Traits (Herberstein et al. 2022) and Elton Traits (Wilman et al. 2014). Additionally, we acquired body mass data using R packages *rfishbase* and *rsealifebase* (Boettiger et al. 2023). We successfully acquired 82% of the species body masses in our dataset (*n* = 1757 of 2152). Next, we inferred locomotion mode from class, family or species names. We used family names if the class column was empty, which was the case for fish families (*n* = 29). We then used species names in two scenarios, (1) if the family was not present (*n* = 8) or (2) if the class corresponded to the wrong locomotion mode, for example, running birds or swimming mammals/birds (*n* = 30). We filled in locomotion modes for all species in our dataset. However, due to sample size limitations, we filtered distance data to only include flying birds, running mammals and swimming fishes. We adapted the underlying allometric scaling relationships of the bioenergetic dispersal model to match these locomotion modes and related taxonomic groups, as shown in Table 1. This resulted in a dataset of 1571 observations, taken from 7 metastudies and 698 empirical studies across a pool of 834 species from 180 taxonomic family groups, spanning 6 orders of magnitude in body mass and 7 orders of magnitude in distance. For an overview of the metastudies used to obtain maximum dispersal distance data and subsequent reference lists, see Appendix S2; Table S2.

#### 2.2.2 Analyses

To assess the influence of body mass on dispersal distances, we standardised our dataset before analyses by converting all body masses to kilograms (kg) and dispersal distances to meters (m). We used the bioenergetic dispersal model parameterized for flying birds, running mammals, and swimming fish to predict the physiological maximum dispersal distances. Theoretical predictions were generated across the full range of empirical body masses. To visually compare the empirical data with model outputs, we plotted the data on a log10 scale and applied a generalised additive model (GAM) for each locomotion mode. The theoretical predictions were then overlaid for a qualitative visual comparison.

All calculations were carried out using R (R Core Team 2021). The following R‐packages were used for the analyses and graphical presentation: tidyverse (Wickham et al. 2019), viridis (Garnier et al. 2024), scales (Wickham et al. 2023), igraph (Csárdi et al. 2024), and ggraph (Pedersen and RStudio 2024). Images used for the figures were sourced from Freepik.com: landscape by brgfx, animal silhouettes by NACreative, Freepik, and macrovector_official, and arrows by juicy_fish. The R package workflowr was used to facilitate reproducibility of model outputs, data analyses, tables, and figures (Blischak et al. 2023). The open‐source code and data are hosted on GitHub [https://github.com/biowilks/Energy‐Budget‐Dispersal‐Model], with the full archived version provided in the repository (Wilkinson et al. 2025).

## 3 Results

### 3.1 Energetics of Dispersal

**FIGURE 3:**
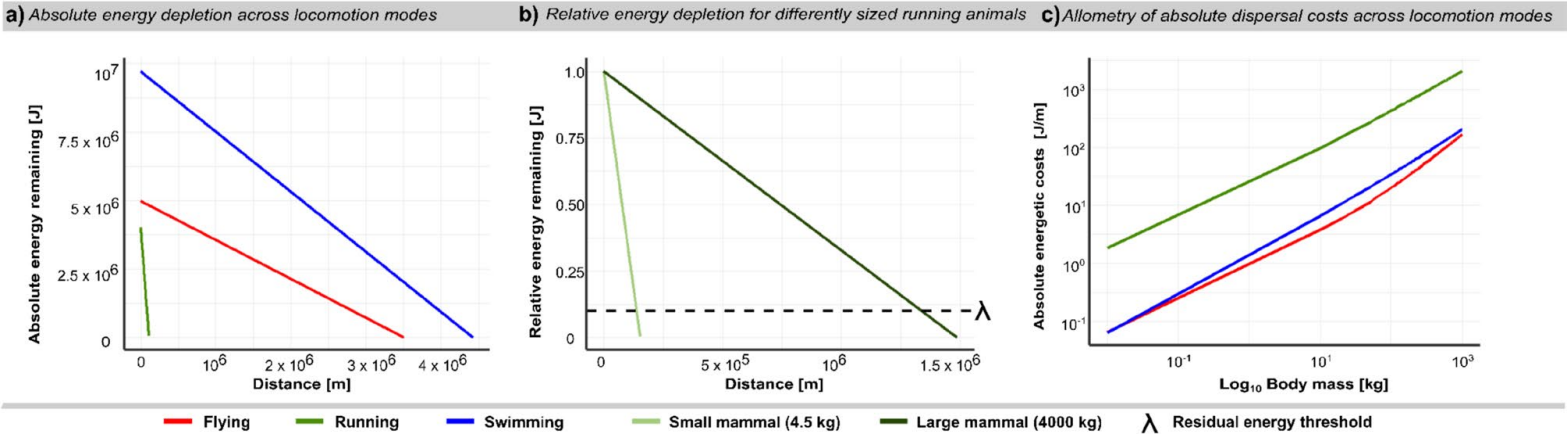
Examples of how to quantify the energetics of animal dispersal. (a) The relationship between absolute energy depletion (i.e., the decay in energy remaining) [J] with dispersal distance [m] for a 2 kg animal across locomotion modes and corresponding taxonomic groups. (b) The relationship between relative energy depletion (i.e., the energetic costs of dispersing relative to the available energy) and distance for differently sized running animals (here depicted for running animals). The steeper slope for the small animal (light green) indicates a lower energetic efficiency compared to the large animal (dark green). The dashed line is a visual representation of lambda λ (here: 0.1), the proportion of energy needed upon arrival for an animal to survive and fulfil its daily tasks. Here, the intersection of both lines with the dashed line shows the maximum dispersal distance of each species respectively. (c) The relationship between absolute cost of dispersal per distance [J/m] with body mass [kg] across locomotion modes and corresponding taxonomic groups.

The bioenergetic dispersal model quantifies a linear absolute energy depletion with dispersal distance across locomotion modes and corresponding taxonomic groups (Figure 3a). The model shows a steeper slope of absolute energy depletion in running compared to flying and swimming animals, but a higher initial energy storage for swimming relative to flying and running animals. This implies that swimming fishes can travel the greatest distances before depleting their energy stores, with running mammals only being able to travel much shorter distances (Figure 3a). Moreover, the model predicts higher energetic efficiency (i.e., the energetic costs of dispersing one unit of distance relative to the available energy) of larger animals compared to smaller animals (Figure 3b) despite the absolute cost of dispersal being higher for larger animals (Figure 3c) (Halsey and White 2017). For instance, a running mammal of 4.5 kg would have absolute energetic costs per distance of dispersal of 58.82 J/m compared to 5390.62 J/m for a 4000 kg mammal (Figure 3c). However, the 4000 kg animal would only use 7% of its available energy to move 100,000 m distance compared to 68% for the 4.5 kg animal (Figure 3b). The absolute energetic costs per unit moved are additionally affected by locomotion mode with running animals having considerably higher costs compared to swimming and flying animals (Schmidt‐Nielsen 1972), which show comparable absolute costs at lower and higher body masses (Figure 3c). Consequently, the bioenergetic model can be used to quantify the species‐specific energetic costs for any given dispersal distance.

**FIGURE 4:**
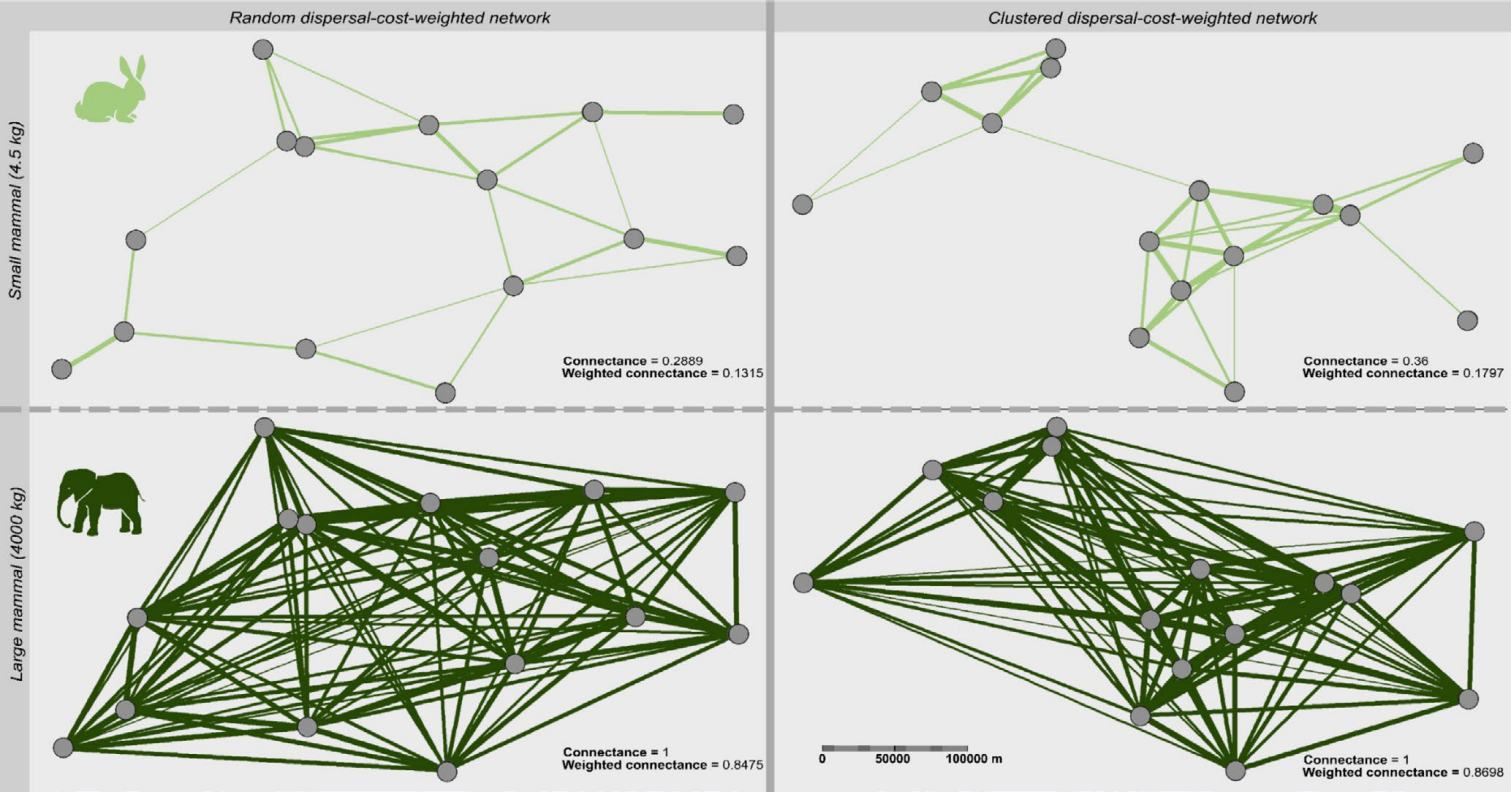
Dispersal‐cost‐weighted spatial networks for differently sized running mammals (4.5 and 4000 kg) across two landscape types (random and clustered). The line thickness indicates the relative energy remaining when travelling from one patch to another, which can be used as an indicator for dispersal flux. For each species and landscape type, the connectance (i.e., based on presence‐absence of dispersal links) and weighted connectance (i.e., based on energetic‐cost‐weighted dispersal links) are shown.

The dispersal‐cost‐weighted spatial networks revealed distinct patterns of landscape connectivity across differently‐sized running mammals and landscape types (Figure 4). Within both random and clustered spatial networks, the line thickness represents the relative energy remaining when travelling from one patch to another, where thicker lines represent more energy remaining in the animal upon arrival (arising from lower energetic costs and indicating higher dispersal flux), while thinner lines indicate lower dispersal fluxes. The absence of a line between patches shows that these patches are too distant from each other and the species is unable to disperse between them. For the large mammal (4000 kg), the random as well as the clustered landscape had a connectance of 1, showing all potential dispersal links were realised within this network. For the small mammal (4.5 kg), the random landscape had a connectance of 0.29, while in the clustered landscape the connectance was slightly higher (0.36). Similarly, the weighted connectance in the random landscape was 0.13 for the small and 0.85 for the large animal, and in the clustered landscape 0.18 for the small and 0.87 for the large animal. Overall, the smaller animal had lower connectance in both landscape types, with 71% less realised links in the random and 64% less realised links in the clustered landscape. Similarly, the small animal has lower weighted connectance in both landscapes compared to the large animal, indicating that it needs approximately 6.5 times more energy to disperse in the random and approximately 5 times more energy to disperse in the clustered landscape than the large animal.

### 3.2 Maximum Dispersal Distance

**FIGURE 5:**
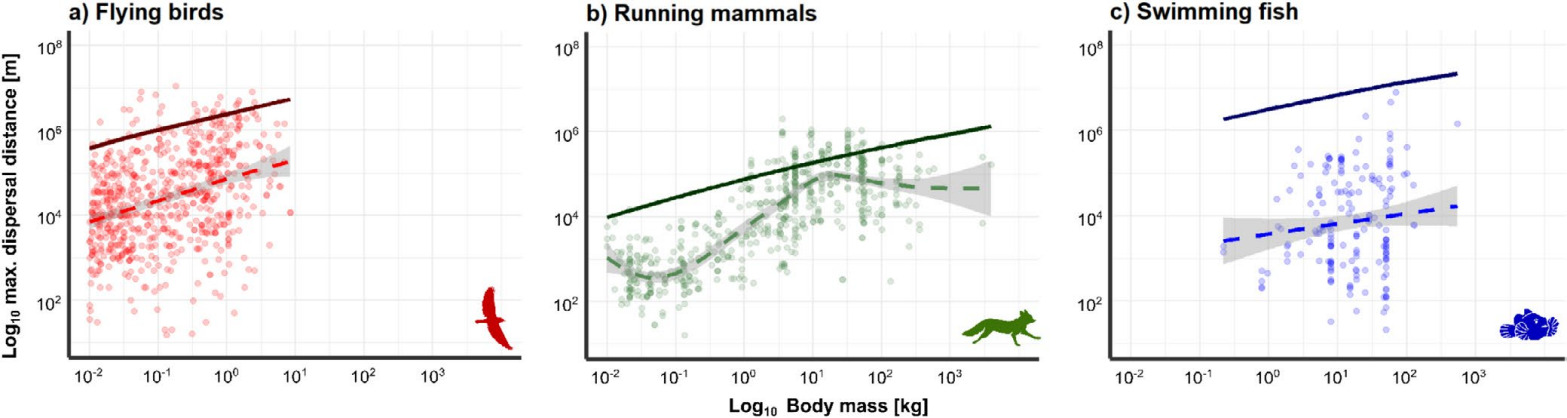
The relationship between maximum dispersal distance and body mass: (a) flying birds, (b) running mammals and (c) swimming fishes. The solid lines represent the absolute maximum dispersal distances predicted using the bioenergetic model for each locomotion mode and related taxonomic group. The dashed line and confidence bands represent the generalised additive model (GAM) output of the empirical data for flying birds (*n* = 744), running mammals (*n* = 648) and swimming fishes (*n* = 179). Note that the GAM is meant as a visual guidance for qualitative comparisons with the model prediction.

We used our bioenergetic model to predict the physiological maximum dispersal distances based on species and their traits. The model predicts an increase in physiological maximum dispersal distance across body mass for all locomotion modes (Figure 5a–c). The GAM fitted to the empirical data also shows a generally positive relationship between realised maximum dispersal distance and body mass in empirical data of flying birds, running mammals, and swimming fishes.

Using the same body mass range as the empirical data (0.0031–8.44 kg), the predicted physiological maximum distance for flying birds ranges from 193.82 to 5435.39 km and 4.4% of the empirical data lies above the model prediction while 95.6% lies below. For running mammals, we predict a maximum distance range of 8.47 to 1332.97 km across body masses (0.0076–3940.0 kg) and 11.6% of the empirical data lies above the model prediction while 88.4% lies below. For swimming fishes, predicted maximum dispersal distances ranged from 1860.75 to 21472.13 km across body masses (0.22–550 kg) and 0% of the empirical data lies above the model prediction while 100% lies below.

## 4 Discussion

We developed a mechanistic dispersal model, incorporating bioenergetics and traits, that quantifies energetic costs of dispersal and predicts physiological maximum dispersal distances of species across locomotion modes and ecosystems.

### 4.1 Energetics of Dispersal

It is known that body size and locomotion mode are important drivers of dispersal capacities (Bowler and Benton 2005; Clobert et al. 2009), and it is not surprising that larger and more mobile organisms generally disperse further. However, quantifying the energetic costs and consequences of dispersal remains challenging. Here, we combine fundamental allometric relationships of ecological processes to quantify these intuitive differences among animals, enabling estimation of energy costs and depletion across distances, locomotion modes, and body sizes (Figure 3). The maximum possible dispersal distance, or physiological maximum, is determined by the energetics of dispersal and represents the point at which an animal's energy reserves are depleted. This energetic quantification is crucial for understanding the feasibility of dispersal events, their associated costs, and their consequences for spatial processes and patterns. As shown in Figure 4, the bioenergetic dispersal model can be used to quantify the energetics within fragmented or patchy landscapes of various configurations across body sizes and locomotion modes. While we presented this application generically, this approach can be applied to real landscapes with known structures and distances.

Additionally, the model can help assess potential colonisation success by quantifying the remaining energy upon arrival, with greater energy reserves indicating a better ability to forage and find mates. Therefore, the bioenergetic dispersal model can enhance the understanding of quantitative colonisation processes by shifting from random or species‐specific colonisation probabilities to trait‐based probabilities and quantitative dispersal fluxes driven by energy availability (see example given in Figure 4). Colonisation plays a crucial role for predicting patterns and dynamics in metacommunities (Thompson et al. 2020) and island‐biogeography (Macarthur and Wilson 1967). The bioenergetic dispersal model could, for instance, enhance modern island‐biogeography models that consider body mass and trophic interactions on islands (Gravel et al. 2011; Jacquet et al. 2017). Moreover, in combination with the energetic processes described by local trophic interactions, this could lead to substantial improvements of meta‐foodweb approaches (Ryser et al. 2019, 2021).

### 4.2 Maximum Dispersal Distance

Empirical data on the energetics of dispersal is challenging to obtain and has only been measured in a limited number of studies (Benoit et al. 2020; Klarevas‐Irby et al. 2021), whereas distance data are more readily available. We compiled maximum dispersal distances for flying, running, and swimming animals, and compared them to predictions from the bioenergetic dispersal model. We expect the model to predict a scaling pattern across body size similar to that observed in empirical data. However, since most empirically measured ‘maximum’ dispersal distances do not represent the absolute physiological limits, we anticipate that most data points will fall below the model's predictions. Consistent with the model prediction, empirical dispersal distances indicate a generally positive scaling with body size (e.g., Jenkins et al. 2007; Sutherland et al. 2000; Whitmee and Orme 2013). We found that 88.4%–100% of the empirical data on maximum dispersal distances fall below the model prediction. This can be due to either methodological or ecological reasons. First, there may be an underestimation of distance in empirical data due to limitations when measuring the maximum dispersal of species. Measurements of distance travelled relies on intermittent location data and assumes straight‐line distances between locations to give a total distance (Rowcliffe Marcus et al. 2012). This may underestimate the true distances travelled as the actual paths taken between locations are not in fact straight (Rowcliffe Marcus et al. 2012). Second, shorter measured maximum dispersal distances may be due to other ecological factors. For instance, the type of dispersal (e.g., natal or breeding) has been shown to affect dispersal distances in birds (Paradis et al. 1998). Also, animals likely stop dispersing when reaching a suitable habitat patch or finding a mate (Clobert et al. 2009; Matthysen 2012). Moreover, physical or climatic barriers might hinder animals from dispersing further (Caplat et al. 2016).

While most data points met our expectation of falling below the model's predictions, some exceeded them for running and flying animals. This can be due to specific traits and adaptations not captured by the model, such as differences in starvation tolerance (e.g., the animals' ability to cope with prolonged periods of food scarcity) (see McCue 2010) and more specific locomotion modes (e.g., soaring flight reducing energy costs or different gaits altering movement speed) (Biewener et al. 2018; Weber 2009), or environmental factors (e.g., wind, precipitation or temperature affecting movement costs) (Kuussaari et al. 2016; Shaw and Kelly 2013; Walls et al. 2005). Additionally, for some methods, potential stop‐overs and re‐fuelling cannot be ruled out (e.g., data from ringing) (see Model Limitations and Extensions). For swimming fishes, all data points fall below the bioenergetic dispersal model prediction and the data average lies 2–3 orders of magnitude below the physiological maximum (as opposed to ~1 order of magnitude for running and flying). The higher maximum distances predicted by the model for swimming compared to flying and running predominantly stems from the empirically supported lower metabolic costs of fishes and the lower locomotion costs of swimming animals (Figure 2b–c). However, the available dispersal data is predominantly from riverine fish species (*n* = 61/66) (Comte and Olden 2018), which may explain the shorter empirical distances observed. The structure of rivers likely imposes constraints on dispersal due to artificial barriers like dams (Zarri et al. 2022) and natural obstacles such as waterfalls or the physical connectivity of the river network (Tonkin et al. 2018). While the model predictions for maximum dispersal of riverine fishes should be interpreted cautiously, the points closest to the model predictions, are, in fact sharks, hinting at a better model fit for open water systems. To further test our maximum dispersal distance predictions, data from fish in less obstructed systems, such as marine environments, would be necessary.

While empirical data on maximum dispersal distances typically represent the realised dispersal of animals, the model provides a context‐independent physiological limit to dispersal distance derived from dispersal energetics, which is relevant to various ecological fields. For instance, while long‐distance dispersal events can strongly influence population dynamics and range expansions, their frequency and magnitude tend to be poorly characterised by empirical dispersal kernels (Nathan et al. 2003; Travis et al. 2013). This necessitates a mechanistic approach toward estimating dispersal kernels that can accommodate multiple underlying processes (Nathan et al. 2012). The bioenergetic model contributes to this goal by providing a biologically feasible estimate of maximum dispersal distance. By informing empirical dispersal kernels with this maximum distance, the model can enhance estimation of the probability distribution of dispersal distances. Dispersal kernels are applied in diverse ecological and evolutionary models, including partial differential equations (e.g., Kot et al. 1996) and integro‐difference equations (e.g., Neubert et al. 1995; Krkošek et al. 2007). They also play a crucial role in shaping habitat management and conservation policies (e.g., Driscoll et al. 2014; Jongejans et al. 2008; Saura et al. 2014). Therefore, the bioenergetic dispersal model can complement other animal dispersal models and their applications by enhancing the underlying dispersal kernels.

### 4.3 Model Limitations and Extensions

The bioenergetic dispersal model assumes that animals travel at speeds that optimise the trade‐off between minimising energetic costs by sustaining high speeds and effectively dissipating metabolic heat during extended dispersal bouts (Dyer et al. 2023). In addition to using an optimal travel speed, we assume that animals allocate their time solely to dispersal. This may be reasonable under specific circumstances where animals avoid stopovers (for resting or feeding) when crossing large areas of disrupted habitat caused by increased human infrastructure and landscape fragmentation, which ultimately affect the movement, behaviour, and dispersal of species (Haila 2002). A similar movement strategy is known from optimal migration theory (i.e., ‘time‐minimization hypothesis’), which states that some species increase their migration speed and limit their time at stop‐overs to reduce risks in unfamiliar habitats (Alerstam and Lindström 1990). The model is also flexible to include such stop‐overs for resting or hiding from predation. We showed that changing the amount of time allocated to resting only marginally affects the overall energetics of the dispersal phase. In the future, the model could also be extended and combined with foraging models to incorporate feeding, providing a more specific understanding of the dispersal process.

The distance that can be covered by animals also depends on their initial energy storage, however the allometries used to calculate the energy storage in the model are only based on the conversion of adipose tissue to energy (Antoł and Kozłowski 2020; Martin et al. 2017). Fat is commonly used for long‐term energy storage due to its greater energy density compared to carbohydrates (Schmidt‐Nielsen 1997). Since our dispersal model is based on energy budgets, it can be adjusted to account for deviations from these assumptions (e.g., Alerstam and Lindström 1990; Weber et al. 1998). While it shares fundamental principles with existing models of maximum migration distance (Hein et al. 2012), the bioenergetic model offers a complementary approach by focusing on another fundamental movement process. Specifically designed to quantify the energetics of dispersal, the model also enables the prediction of maximum dispersal distances and dispersal‐cost‐weighted spatial networks. However, since our approach assumes straight‐line dispersal, which might not always reflect natural movement patterns, it might overestimate the distances animals can disperse and, therefore, the actual landscape connectivity. Another promising avenue would be to integrate the energetics of dispersal, provided by the model, in the framework of energy landscapes (Berti et al. 2022; Shepard et al. 2013) to connect animal energy budgets with the environmentally‐driven cost of moving through different physical environments (Halsey and White 2017).

## 5 Outlook

Here, we developed a bioenergetic dispersal model capable of estimating the energetics and limits of dispersal. The integration of this model into ecological and evolutionary approaches constitutes a promising avenue to further our fundamental understanding across local community ecology, landscape ecology, and biogeography under global change.

For instance, anthropogenic impacts have important implications for animal movement (Spiegel et al. 2017; Tucker et al. 2018; Wilson et al. 2020). Habitat fragmentation can reduce the availability and suitability of adjacent habitats and increase the distances between suitable habitat patches (Mullu 2016), leading to isolation of populations. The bioenergetic dispersal model can be used to assess the costs and feasibility of connecting suitable habitat fragments in real landscapes based on an animal's body size and locomotion mode. This can provide insights into how well landscapes are connected depending on the species in focus (Hirt et al. 2018), which can be important for conservation efforts (e.g., location of wildlife corridors within protected area networks) (Santini et al. 2016). Furthermore, the model can inform (re‐)colonisation success based on dispersal‐cost‐weighted spatial networks, species distributions and range dynamics, and thereby have important implications for gene‐flow and speciation or the spread of diseases (Garant et al. 2007; Sumner et al. 2017). Finally, in the face of ongoing climate change, integrating the temperature‐dependence of the processes underlying the bioenergetic dispersal model can help better understand animal dispersal capacities under different temperature regimes.

Overall, the model holds relevance for researchers in the field of ecology and evolution, conservation practitioners, and policymakers alike. Ultimately, a mechanistic and predictive understanding of animal dispersal will enhance our knowledge of landscape connectivity, species distributions, and biodiversity patterns in a changing world.

## Author Contributions

Conceptualisation: C.W., M.R.H., R.R., U.B., A.D. Formal analysis: C.W., M.R.H., R.R. Writing original draft: C.W., M.R.H., R.R. Writing review and editing: C.W., M.R.H., R.R., U.B., A.D. Visualisation: C.W., M.R.H., R.R., A.D. Funding acquisition: U.B.

### Peer Review

The peer review history for this article is available at https://www.webofscience.com/api/gateway/wos/peer‐review/10.1111/ele.70085.

## Supporting information


Data S1.

## Data Availability

The open‐source code and data used for the analyses are available on GitHub at https://github.com/biowilks/Energy‐Budget‐Dispersal‐Model. Additional details on the structure of the analysis are provided at https://biowilks.github.io/Energy‐Budget‐Dispersal‐Model and the full archived version provided in the repository https://doi.org/10.5281/zenodo.14717650 (Wilkinson et al. 2025).
