# Supplementary material for: A Mechanistic Approach to Animal Dispersal—Quantifying Energetics and Maximum Distances
Source: Ecol Lett. 2025 Feb 20;28(2):e70085. doi: 10.1111/ele.70085 (PMC11840884; doi:10.1111/ele.70085)
Supplement: Supplementary file 1 — Data S1. [file ELE-28-0-s001.docx]

**SUPPLEMENTARY INFORMATION**

**A mechanistic approach to animal dispersal - quantifying energetics and maximum distances**

Caitlin Wilkinson, Ulrich Brose, Alexander Dyer, Myriam R. Hirt, Remo Ryser

**This file includes**

**Table S1.** Conversion factors and equations underlying the bioenergetic dispersal model.

**Figure S1.** Sensitivity analyses showing the effect of changing the residual energy needed upon arrival (𝝀) on maximum dispersal distance predictions.

**Figure S2.** Sensitivity analyses showing the effect of adding resting time or stop overs (ꞵ) on maximum dispersal distance predictions.

**Table S2.** Overview of the number of datapoints and empirical references from each metastudy the maximum dispersal distance data was obtained from.

**Appendix S2.** Full list of references used to obtain maximum dispersal distance data.

**Table S1.** Overview of parameters, reported values and units as provided in the original references, including the conversion factors and equations necessary for applying these values to the bioenergetic dispersal model. This table provides a comprehensive resource for understanding how the original data has been adapted to support the model's calculations.

| **a) Conversion factors** | | | | | | |
| --- | --- | --- | --- | --- | --- | --- |
|  | | Value | | | Transformation | Reference |
| $ɣ_{1}$ | | $40 \cdot{10}^{6}$ | | | $kg$ → $J$ | Peters. 1986 |
| $ɣ_{2}$ | | $1000$ | | | $kJ$ → $J$ | NA |
| $ɣ_{3}$ | | $20$ | | | $ml O_{2}h^{-1}$ → ${J h}^{-1}$ | Peters. 1986 |
| $ɣ_{4}$ | | $3600$ | | | ${J h}^{-1}$ → ${J s}^{-1}$ | NA |
| $ɣ_{5}$ | | $1.43$ | | | $ml O_{2}$ → $mg O_{2}$ | Peters. 1986 |
| $ɣ_{6}$ | | $1000$ | | | $kg$ → $g$ | NA |
| **b) Equations underlying bioenergetic dispersal model** | | | | | | |
| Output | Group | Parameter | Reported value | Reported units | Conversion equation | Reference |
| Energy storage [$J$] | Birds | $a_{1}$ | $0.06$ | ${{kg kg}^{-b_{1}}}$ | $a_{1}{\cdotɣ}_{1}$ | Antol & Kozlowski. 2020 |
|  |  | $b_{1}$ | $0.98$ | $NA$ | $b_{1}$ |  |
|  | Mammals | $a_{2}$ | $0.05$ | ${kg kg}^{-b_{2}}$ | $a_{2}{\cdotɣ}_{1}$ |  |
|  |  | $b_{2}$ | $1.00$ | $NA$ | $b_{2}$ |  |
|  | Fish | $a_{3}$ | $4.17$ | ${kJ g}^{-b_{3}}$ | $a_{3}{\cdotɣ}_{2}\cdot(\frac{1}{{ɣ_{6}}^{b_{3}+1}})$ | Martin et al. 2017 |
|  |  | $b_{3}$ | $0.02$ | $NA$ | $b_{3}+ 1$ |  |
| Basal metabolic rate  [${J h}^{-1}$] | Birds | $a_{4}$ | $7.43$ | ${ml O_{2}h^{-1} g}^{-b_{4}}$ | $a_{4}{\cdotɣ}_{3}{{\cdotɣ}_{6}}^{b_{4}} \cdot(\frac{1}{ɣ_{4}})$ | Gavrilov et al. 2022 |
|  |  | $b_{4}$ | $0.65$ | $NA$ | $b_{4}$ |  |
|  | Mammals | $a_{5}$ | $3.25$ | ${ml O_{2}h^{-1} g}^{-b_{5}}$ | $a_{5}{\cdotɣ}_{3}{{\cdotɣ}_{6}}^{b_{5}} \cdot(\frac{1}{ɣ_{4}})$ |  |
|  |  | $b_{5}$ | $0.74$ | $NA$ | $b_{5}$ |  |
|  | Fish | $a_{6}$ | $74.13$ | ${mg O_{2}h^{-1} kg}^{-b_{6}}$ | $a_{6}\cdot(\frac{1}{ɣ_{5}}){\cdotɣ}_{3}\cdot(\frac{1}{ɣ_{4}})$ | Watanabe & Payne. 2023 |
|  |  | $b_{6}$ | $0.95$ | $NA$ | $b_{6}$ |  |
| Travel speed  [$m s^{-1}$] | Flying | $v_{0fly}$ | $30.54$ | ${m s^{-1} kg}^{-c}$ | $v_{0fly}$ | Dyer et al. 2023 |
|  | Running | $v_{0run}$ | $0.28$ | ${m s^{-1} kg}^{-c}$ | $v_{0run}$ |  |
|  | Swimming | $v_{0swim}$ | $0.39$ | ${m s^{-1} kg}^{-c}$ | $v_{0swim}$ |  |
|  | All | $c$ | $0.27$ | $NA$ | $c$ |  |
|  |  | $k$ | $0.033$ | ${s m^{-1} kg}^{-c}$ | $k$ |  |
|  |  | $d$ | $0.24$ | $NA$ | $d$ |  |
| Locomotion costs [${J s}^{-1}$] | Flying | $a_{7}$ | $32.00$ | ${J s^{-1} m^{-b_{8}} kg}^{-b_{7}}s^{-b_{8}}$ | $a_{7}$ | Tucker 1973 in Peters 1986 (Chapter 6) |
|  |  | $a_{8}$ | ${3.30 x 10}^{-3}$ | ${J s^{-1} m^{-b_{9}} kg}^{-b_{7}}s^{-b_{9}}$ | $a_{8}$ |  |
|  |  | $a_{9}$ | ${5.80 x 10}^{-3}$ | ${J s^{-1} m^{-b_{9}} kg}^{-b_{10}}s^{-b_{9}}$ | $a_{9}$ |  |
|  |  | $b_{7}$ | $-0.34$ | $NA$ | $b_{7}+1$ |  |
|  |  | $b_{8}$ | -$1.00$ | $NA$ | $b_{8}$ |  |
|  |  | $b_{9}$ | $2.50$ | $NA$ | $b_{9}$ |  |
|  |  | $b_{10}$ | -$0.51$ | $NA$ | $b_{10}+1$ |  |
|  | Running | $a_{10}$ | $11.30$ | ${J s^{-1} m^{-1} kg}^{-b_{11}}s^{-1}$ | $a_{10}$ | Peters 1986 (Chapter 6) |
|  |  | $b_{11}$ | $-0.36$ | $NA$ | $b_{11}+1$ |  |
|  | Swimming | $a_{11}$ | $0.116$ | $J s^{-1}$ | $a_{11}$ | Beamish 1978 in Peters 1986 (Chapter 6) |
|  |  | $a_{12}$ | $1.88$ | $J s^{-1}$ | $a_{12}$ |  |
|  |  | $b_{13}$ | $-0.36$ | $NA$ | $b_{13}$ |  |
| Field metabolic rate [${J s}^{-1}$] | Flying | $a_{13}$ | $1.1$0 | $NA$ | $a_{13}$ | Peters. 1986  (Chapter 6) |
|  | Running | $a_{14}$ | $1.20$ | $NA$ | $a_{14}$ |  |

**
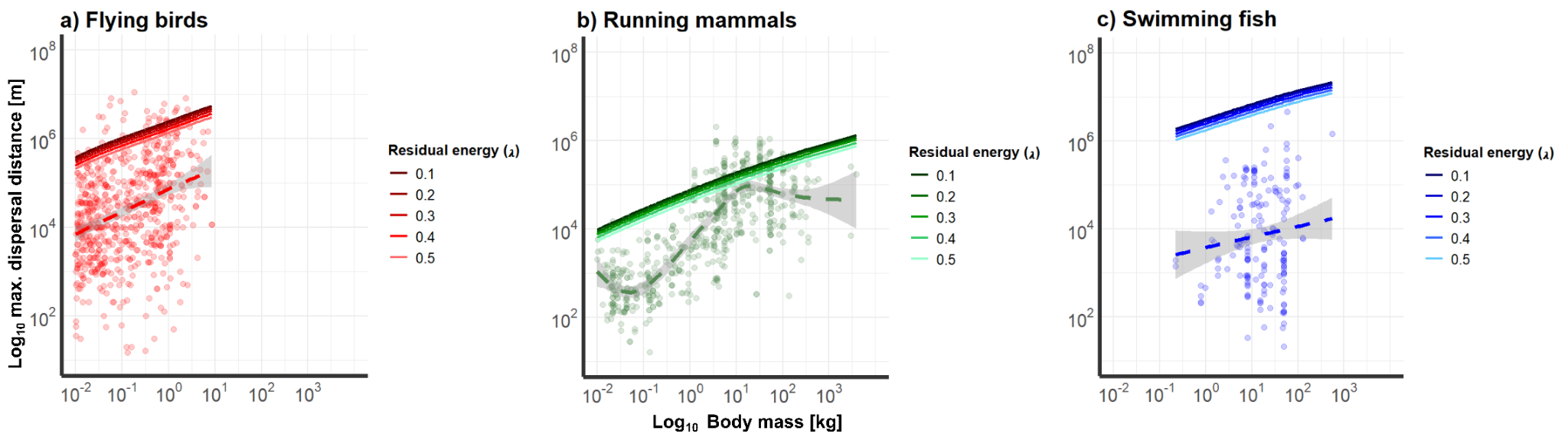
**

**Figure S1. Sensitivity analyses showing the effect of changing the residual energy needed upon arrival (𝝀) on maximum dispersal distance predictions:** a) flying birds, b) running mammals and c) swimming fishes. The solid lines represent the absolute maximum dispersal distances predicted using the bioenergetic model for each locomotion mode and related taxonomic group. The dashed line and confidence bands represent the generalised additive model (GAM) output of the empirical data for flying birds (n = 744), running mammals (n = 648) and swimming fishes (n = 179). Note that the GAM is meant as a visual guidance for qualitative comparisons with the model prediction.

**
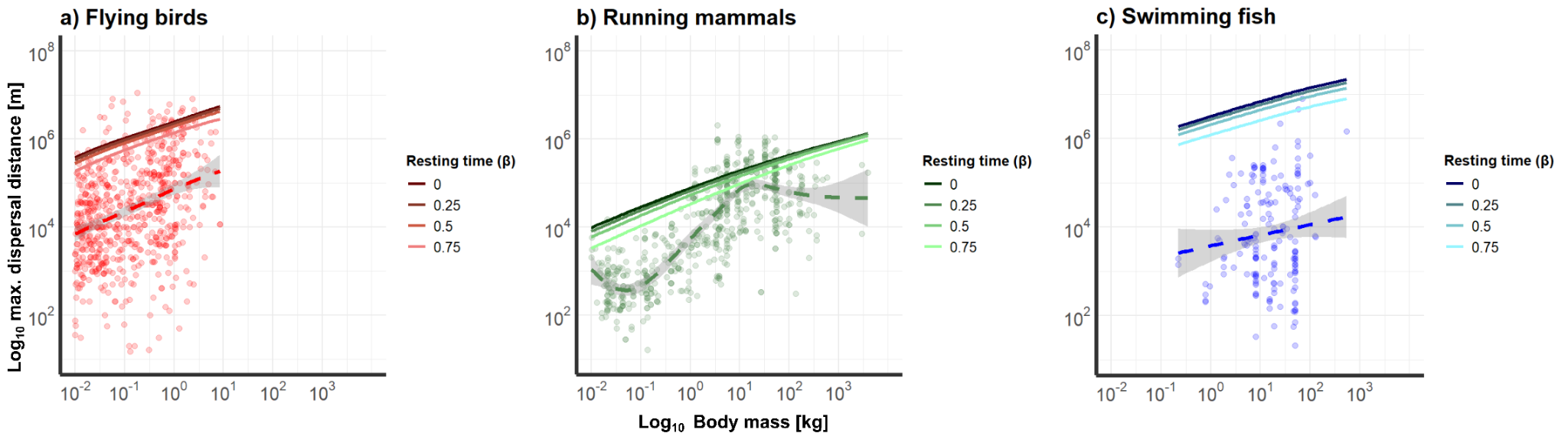
**

**Figure S2. Sensitivity analyses showing the effect of adding resting time or stop overs (ꞵ) on maximum dispersal distance predictions:** a) flying birds, b) running mammals, and c) swimming fishes. The solid lines represent the absolute maximum dispersal distances predicted using the bioenergetic model for each locomotion mode and related taxonomic group. The dashed lines and confidence bands represent the generalised additive model (GAM) output of the empirical data for flying birds (n = 744), running mammals (n = 648), and swimming fishes (n = 179). The GAM is included as a visual aid for qualitative comparisons with the model predictions.

**Table S2.** Overview of the number of datapoints and empirical references from each metastudy the maximum dispersal distance data was obtained from. Note that the total number of empirical references is less than the combined total of empirical references for each group, as some references appear in multiple metastudies.

| **Group** | **Metastudy** | **Datapoints (n)** | **Empirical references (n)** |
| --- | --- | --- | --- |
| *Flying bird* | Sutherland et al. 2000 | 117 | 71 |
|  | Stewart et al. 2022 | 296 | 37 |
|  | BirdLife International | 245 | 205 |
|  | Straus et al. 2023 | 80 | 1 |
|  |  | Total: 744 | Total: 290 |
| *Running mammal* | Whitmee & Orme. 2013 | 178 | 88 |
|  | Sutherland et al. 2000 | 87 | 53 |
|  | Santini et al. 2013 | 370 | 255 |
|  | Straus et al. 2023 | 13 | 1 |
|  |  | Total: 648 | Total: 329 |
| *Swimming fish* | Olden. 2020 | 174 | 81 |
|  | Straus et al. 2023 | 5 | 1 |
|  |  | Total: 179 | Total: 82 |

**Appendix S2.** Full list of references used to obtain maximum dispersal distance data.

**Metastudies:**

BirdLife International. (unpublished). Bird dispersal distance estimates dataset. Data available upon request from [https://datazone.birdlife.org/species/requestdis].

Olden, J. (2020). Comte, L. and J.D. Olden. 2018. Fish dispersal in flowing waters: a synthesis of movement- and genetic-based studies. Fish and Fisheries 19: 1063-1077.. figshare. Dataset. https://doi.org/10.6084/m9.figshare.5711401.v1

Santini, L., Marco, M.D., Visconti, P., Baisero, D., Boitani, L. & Rondinini, C. (2013). Ecological correlates of dispersal distance in terrestrial mammals. Hystrix It. J. Mamm., 24, 181–186.

Stewart, P.S., Voskamp, A., Santini, L., Biber, M.F., Devenish, A.J.M., Hof, C., et al. (2022). Global impacts of climate change on avian functional diversity. Ecology Letters, 25, 673–685.

Straus, S., Forbes, C., Little, C.J., Germain, R.M., Main, D.A., O’Connor, M.I., et al. (2024). Macroecological constraints on species’ ‘movement profiles’: Body mass does not explain it all. Global Ecology and Biogeography, 33, 227–243.

Sutherland, G.D., Harestad, A.S., Price, K. & Lertzman, K.P. (2000). Scaling of Natal Dispersal Distances in Terrestrial Birds and Mammals. Conservation Ecology, 4.

Whitmee, S. & Orme, C.D.L. (2013). Predicting dispersal distance in mammals: a trait-based approach. Journal of Animal Ecology, 82, 211–221.

**Flying bird:**

Adamcik, R.S. & Keith, L.B. (1978). Regional movements and mortality of great horned owls in relation to snowshoe hare fluctuations. Can. Field-Nat., 92, 228–234.

Adkisson, C.S. (1999). Personal communication to BirdLife International (in litt.).

Aebischer, N.J. (1995). Philopatry and colony fidelity of Shags Phalacrocorax aristotelis on the east coast of Britain. Ibis, 137, 11–18.

Ailes, I.W. (1976). Ecology of the Upland Sandpiper in Central Wisconsin. Master's Thesis, University of Wisconsin.

Aldous, S.E. (1942). The White-necked Raven in relation to agriculture. United States Fish and Wildlife Service.

Allen, R.W. & Nice, M.M. (1952). A study of the breeding biology of the purple martin (Progne subis). Am. Midl. Nat., 47, 606–645.

Anders, A.D., Faaborg, J. & Thompson, F. III (1998). Postfledging dispersal, habitat use, and home-range size of juvenile wood thrushes. Auk, 115, 349–358.

Andersson, M. (1981). Reproductive tactics of the long-tailed skua Stercorarius longicaudus. Oikos, 37, 287–294.

Arendt, W.J. & Vargas Mora, T.A. (1984). Range expansion of the Shiny Cowbird in the Dominican Republic. J. Field Ornithol., 55, 104–107.

Atwood, J.L. & Lerman, S.B. (2006). Family Polioptilidae (Gnatcatchers). In Handbook of the Birds of the World Volume 11: Old World Flycatchers to Old World Warblers (Lynx Edicions, Barcelona).

Austin, J.E., Custer, C.M. & Afton, A.D. (1998). Lesser Scaup. In The Birds of North America Online (A. Poole ed.). Cornell Laboratory of Ornithology, Ithaca.

Badyaev, A.V., Gibson, D.D. & Kessel, B. (1996). White Wagtail, Montacilla alba. In A. Poole & F. Gill (Eds.), The Birds of North America (No. 236/237). The Birds of North America, Inc., Philadelphia, Pennsylvania, USA.

Baker, H. & Baker, P.E. (2000). Maui Alauahio. In The Birds of North America Online (A. Poole ed.). Cornell Laboratory of Ornithology, Ithaca.

Baker-Gabb, D.J. (1996). Family Pedionomidae (Plains-wanderer). In Handbook of the Birds of the World Volume 3: Hoatzin to Auks (J. del Hoyo, A. Elliott & J. Sargatal eds). Lynx Edicions, Barcelona.

Banko, P.C., Ball, D.L. & Banko, W.E. (2002). Hawaiian Crow. In The Birds of North America Online (A. Poole ed.). Cornell Laboratory of Ornithology, Ithaca.

Banko, P.C., Black, J.M. & Banko, W.E. (1999). Hawaiian Goose. In The Birds of North America Online (A. Poole ed.). Cornell Laboratory of Ornithology, Ithaca.

Baptista, L.F., Trail, P.W. & Horblit, H.M. (1997). Family Columbidae (Pigeons and Doves). In Handbook of the Birds of the World Volume 4: Sandgrouse to Cuckoos (J. del Hoyo, A. Elliott & J. Sargatal eds). Lynx Edicions, Barcelona.

Barlow, J.C., Leckie, S.N. & Baril, C.T. (1999). Gray Vireo. In The Birds of North America Online (A. Poole ed.). Cornell Laboratory of Ornithology, Ithaca.

Beaudette, P.D. & Keppie, D.M. (1992). Survival of dispersing spruce grouse. Can. J. Zool., 70, 693–697.

Bednarz, J.C. (1995). Harris' Hawk, Parabuteo unicinctus. In A. Poole & F. Gill (Eds.), The Birds of North America (No. 146). Academy of Natural Sciences, Philadelphia, Pennsylvania, USA, and American Ornithologists' Union, Washington, D.C., USA.

Belson, M.S. (1998). Red-headed Woodpecker (Melanerpes erythrocephalus) use of habitat at Wekiwa Springs State Park, Florida. Master's Thesis, University of Central Florida.

Belthoff, J.R. & Ritchison, G. (1989). Natal dispersal of Eastern Screech-Owls. Condor, 91, 254–265.

Berndt, R. & Sternberg, H. (1968). Terms, studies and experiments on the problems of bird dispersion. Ibis, 110, 256–269.

Beveridge, O.S. (2012). Personal communication to BirdLife International (in litt.).

Bibles, B.D., Glinski, R.L. & Johnson, R.R. (2002). Gray Hawk. In The Birds of North America Online (A. Poole ed.). Cornell Laboratory of Ornithology, Ithaca.

Blackmore, C.J., Peakall, R. & Heinsohn, R. (2011). The absence of sex-biased dispersal in the cooperatively breeding grey-crowned babbler. J. Anim. Ecol., 80, 69–78.

Blums, P., Nichols, J.D., Hines, J.E., Lindberg, M.S. & Mednis, A. (2003). Estimating natal dispersal movement rates of female European ducks with multistate modelling. J. Anim. Ecol., 72, 1027–1042.

Bock, C.E. (1970). The ecology and behavior of the Lewis's Woodpecker (Asyndesmus lewis). Univ. Calif. Publ. Zool., 92, 1–100.

Bowman, R. (2002). Common Ground-dove. In The Birds of North America Online (A. Poole ed.). Cornell Laboratory of Ornithology, Ithaca.

Boydstun, C.P. (1982). Evaluations of the current status of white-fronted doves in South Texas. Master's Thesis, Texas A&M Univ., Kingsville.

Brooker, L.C. & Brooker, M.G. (2002). Dispersal and population dynamics of the blue-breasted fairy-wren, Malurus pulcherrimus, in fragmented habitat in the Western Australian wheatbelt. Wildl. Res., 29, 225–233.

Brown, P.W. & Fredrickson, L.H. (1997). White-winged Scoter. In The Birds of North America Online (A. Poole ed.). Cornell Laboratory of Ornithology, Ithaca.

Brown, D.E., Hagelin, J.C., Taylor, M. and Galloway, J. (1998) Gambel's Quail. In The Birds of North America Online (A. Poole ed.). Cornell Laboratory of Ornithology, Ithaca.

Bryan, D.C. (1996). Family Aramidae (Limpkin). In Handbook of the Birds of the World Volume 3: Hoatzin to Auks (J. del Hoyo, A. Elliott & J. Sargatal eds). Lynx Edicions, Barcelona.

Bull, E.L. & Jackson, J.A. (1995). Pileated Woodpecker, Dryocopus pileatus. In A. Poole & F. Gill (Eds.), The Birds of North America (No. 148). Academy of Natural Sciences, Philadelphia, Pennsylvania, USA, and American Ornithologists' Union, Washington, D.C., USA.

Bull, E.L. & Duncan, J.R. (1993). Great Gray Owl, Strix nebulosa. In A. Poole & F. Gill (Eds.), The Birds of North America (No. 41). Academy of Natural Sciences, Philadelphia, Pennsylvania, USA, and American Ornithologists' Union, Washington, D.C., USA.

Bull, E.L. & Collins, C.T. (1996). Nest site fidelity, breeding age, and adult longevity in the Vaux's Swift. North Am. Bird Bander, 21, 49–51.

Burness, G.P., Lefevre, K. & Collins, C.T. (1999). Elegant Tern. In The Birds of North America Online (A. Poole ed.). Cornell Laboratory of Ornithology, Ithaca.

Bustnes, J.O. & Erikstad, K.E. (1993). Site fidelity in breeding Common Eider Somateria mollissima females. Ornis Fennica, 70, 11–16.

Butcher, G.S. (1991). Mate choice in female Northern Orioles with a consideration of the role of the black male coloration in female choice. Condor, 93, 82–88.

Butler, R.G. & Buckley, D.E. (2002). Black Guillemot. In The Birds of North America Online (A. Poole ed.). Cornell Laboratory of Ornithology, Ithaca.

Cadahía, L., López-López, P., Urios, V., Soutullo, A. & Negro, J.J. (2009). Natal dispersal and recruitment of two Bonelli's Eagles Aquila fasciata: A four-year satellite tracking study. Acta Ornithol., 44, 193–198.

Cale, P.G. (2003). The influence of social behaviour, dispersal and landscape fragmentation on population structure in a sedentary bird. Biol. Conserv., 109, 237–248.

Carmen, W.J. (1988). Behavioral ecology of the California Scrub Jay (Aphelocoma coerulescens californica): A non-cooperative breeder with close cooperative relatives. PhD Thesis, University of California.

Carroll, J.P. (1994). Family Odontophoridae (New World Quails). In Handbook of the Birds of the World Volume 2: New World Vultures to Guineafowl (J. del Hoyo, A. Elliott & J. Sargatal eds). Lynx Edicions, Barcelona.

Chapman, L.B. (1955). Studies of a tree swallow colony. Bird-Banding, 26, 45–70.

Christensen, G.C. (1970). The Chukar Partridge: Its introductions, life history and management. Nevada Fish and Game Department.

Christensen, G.C. (1998). Himalayan Snowcock. In The Birds of North America Online (A. Poole ed.). Cornell Laboratory of Ornithology, Ithaca.

Clarkson, K.E. & Laniawe, A.E. (2000). Hawaiian Hawk. In The Birds of North America Online (A. Poole ed.). Cornell Laboratory of Ornithology, Ithaca.

Collar, N.J. (1997). Family Psittacidae (Parrots). In Handbook of the Birds of the World Volume 4: Sandgrouse to Cuckoos (J. del Hoyo, A. Elliott & J. Sargatal eds). Lynx Edicions, Barcelona.

Collar, N.J. & Newton, I. (2010). Family Fringillidae (Finches). In Handbook of the Birds of the World Volume 15: Weavers to New World Warblers (J. del Hoyo, A. Elliott & D.A. Christie eds). Lynx Edicions, Barcelona.

Collister, D.M. & De Smet, K. (1997). Breeding and natal dispersal in the Loggerhead Shrike. J. Field Ornithol., 68, 273–282.

Cooke, M.T. (1946). Wanderings of the Mockingbird. Bird Banding, 17, 784.

Cossee, R.O. (1995). New Zealand Sooty Tern (Sterna fuscata) breeds in the Seychelles. Notornis, 43, 280.

Coulson, J.C. & Nèves de Mérignies, G. (1992). Where do young Kittiwakes Rissa tridactyla breed, philopatry or dispersal? Ardea, 80, 187–197.

Crocoll, S.T. (1994). Red-shouldered Hawk, Buteo lineatus. In A. Poole & F. Gill (Eds.), The Birds of North America (No. 107). Academy of Natural Sciences, Philadelphia, Pennsylvania, USA, and American Ornithologists' Union, Washington, D.C., USA.

Csada, R.D. & Brigham, R.M. (1994). Breeding biology of the Common Poorwill at the northern edge of its distribution. J. Field Ornithol., 65, 186–193.

Custer, T.W. & Pitelka, F.A. (1977). Demographic features of a Lapland Longspur population near Barrow, Alaska. Auk, 94, 505–525.

Davis, S.K. & Lanyon, W.E. (2008). Western Meadowlark. In The Birds of North America Online (A. Poole ed.). Cornell Laboratory of Ornithology, Ithaca.

De Juana, J. (1994). Family Tetraonidae (Grouse). In Handbook of the Birds of the World Volume 2: New World Vultures to Guineafowl (J. del Hoyo, A. Elliott & J. Sargatal eds). Lynx Edicions, Barcelona.

Dias, R.I. & Macedo, R.H. (2010). Campo Flicker (Colaptes campestris). In Neotropical Birds Online (T.S. Schulenberg ed.). Cornell Laboratory of Ornithology, Ithaca.

Diniz, P. & Santos, E.S.A. (2010). Coal-crested Finch (Charitospiza leucosma). In Neotropical Birds Online (T.S. Schulenberg ed.). Cornell Laboratory of Ornithology, Ithaca.

Dow, H. & Fredga, S. (1983). Breeding and natal dispersal of the Goldeneye, Bucephala clangula. J. Anim. Ecol., 52, 681–695.

Dowling, D.K., Antos, M. & Sahlman, T. (2003). Dispersal and recruitment of juvenile Red-capped Robins, Petroica goodenovii. Emu, 103, 199–205.

Drent, R.H. (1965). Breeding biology of the Pigeon Guillemot Cepphus columba. Ardea, 53, 99–160.

Drilling, N.E. & Thompson, C.F. (1988). Natal and breeding dispersal in House Wrens (Troglodytes aedon). Auk, 105, 480–491.

Dugger, B.D., Dugger, K.M. & Fredrickson, L.H. (1994). Hooded Merganser, Lophodytes cucullatus. In A. Poole & F. Gill (Eds.), The Birds of North America.

Dunk, J.R. (1995). White-tailed Kite, Elanus leucurus. In A. Poole & F. Gill (Eds.), The Birds of North America (No. 178). Academy of Natural Sciences, Philadelphia, Pennsylvania, USA, and American Ornithologists' Union, Washington, D.C., USA.

Dunlop, J.N. & Jenkins, J. (1992). Known-age birds at a subtropical breeding colony of the Bridled Tern (Sterna anaethetus): A comparison with the Sooty Tern. Colonial Waterbirds, 15, 75–82.

Dunnet, G.M., Ollason, J.C. & Anderson, A. (1979). A 28-year study of breeding Fulmars Fulmarus glacialis in Orkney. Ibis, 121, 293–300.

Eaton, S.W. (1992). Wild Turkey, Meleagris gallopavo. In A. Poole & F. Gill (Eds.), The Birds of North America (No. 22). Academy of Natural Sciences, Philadelphia, Pennsylvania, USA, and American Ornithologists' Union, Washington, D.C., USA.

Eden, S.F. (1987). Natal philopatry of the magpie Pica pica. Ibis, 129, 477–490.

Elliott, A. (1992). Family Ciconiidae (Storks). In Handbook of the Birds of the World Volume 1: Ostrich to Ducks (J. del Hoyo, A. Elliott & J. Sargatal eds). Lynx Edicions, Barcelona.

Elliott, A. (1994). Family Megapodiidae (Megapodes). In Handbook of the Birds of the World Volume 2: New World Vultures to Guineafowl (J. del Hoyo, A. Elliott & J. Sargatal eds). Lynx Edicions, Barcelona.

Elorriaga, J., Zuberogoitia, I., Castillo, I., Azkona, A., Hidalgo, S., Astorkia, L., Ruiz-Moneo, F. & Iraeta, A. (2009). First documented case of long-distance dispersal in the Egyptian Vulture (Neophron percnopterus). J. Raptor Res., 43, 142–145.

Ely, C.R. & Dzubin, A.X. (1994). Greater White-fronted Goose. In The Birds of North America Online (A. Poole ed.). Cornell Laboratory of Ornithology, Ithaca.

Emfinger, J.W. (1966). Survival, dispersal and reproductive success of the Black Francolin (Francolinus francolinus) in Morehouse Parish. Master’s Thesis, Louisiana State University.

Erritzøe, J. (2003). Family Pittidae (pittas). In Handbook of the Birds of the World Volume 8: Broadbills to Tapaculos (J. del Hoyo, A. Elliott, D. Christie & M. Bruce eds). Lynx Edicions, Barcelona.

Evers, D., Kaplan, J.D., Reaman, P.S., Paruk, J.D. & Phifer, P. (2000). A demographic characterization of the Common Loon in the Upper Great Lakes. In Loons: Old History and New Findings (J.W. McIntyre & D. Evers eds). North American Loon Fund, Holderness.

Fang, Y. & Sun, Y.-H. (1997). Brood movement and natal dispersal of Hazel Grouse (Bonasa bonasia) at Changbai Mountain, Jilin Province, China. Wildlife Biol., 3, 261–264.

Ferrer, M. (1993). Ontogeny of dispersal distances in young Spanish Imperial Eagles. Behav. Ecol. Sociobiol., 32, 259–263.

Fisk, E.J. (1974). Wintering populations of Painted Buntings in southern Florida. Bird Banding, 45, 353–359.

Fjeldså, J. (1996). Family Chionidae (Sheathbills). In Handbook of the Birds of the World Volume 3: Hoatzin to Auks (J. del Hoyo, A. Elliott & J. Sargatal eds). Lynx Edicions, Barcelona.

Flint, P.L. & Herzog, M.P. (1999). Breeding of Steller's Eiders, Polysticta stelleri, on the Yukon-Kuskokwim Delta, Alaska. Can. Field-Nat., 113, 306–308.

Flood, N.J. (1980). The adaptive significance of delayed plumage maturation in the Northern Oriole. Master’s Thesis, University of Toronto.

Flood, N.J. (2002). Scott's Oriole. In The Birds of North America Online (A. Poole ed.). Cornell Laboratory of Ornithology, Ithaca.

Flores, R.E. & Eddleman, W.R. (1991). Ecology of the California Black Rail in southwestern Arizona. Arizona Department of Game and Fish.

Fogarty, M.J. & LaHart, D.E. (1971). Florida Duck movements. Proc. Southeast. Assoc. Fish Wildl. Agencies, 25, 191–202.

Folse, L.J. Jr. (1974). Population ecology of Roadrunners (Geococcyx californianus) in south Texas. Master’s Thesis, Texas A&M University.

Forero, M.G., Donázar, J.A., Blas, J. & Hiraldo, F. (1999). Causes and consequences of territory change and breeding dispersal distance in the Black Kite. Ecology, 80, 1298–1310.

Forsman, E.D., Anthony, R.G., Reid, J.A., Loschl, P.J., Sovern, S.G., Taylor, M., Biswell, B.L., Ellingson, A., Meslow, E.C., Miller, G.S., Swindle, K.A., Thrailkill, J.A., Wagner, F.F. & Seaman, D.E. (2002). Natal and breeding dispersal of Northern Spotted Owls. Wildl. Monogr., 149, 1–35.

Fry, C.H. (2001). Family Meropidae (Bee-eaters). In Handbook of the Birds of the World Volume 6: Mousebirds to Hornbills (J. del Hoyo, A. Elliott & J. Sargatal eds). Lynx Edicions, Barcelona.

Gabrey, S.W. (1996). Migration and dispersal in Great Lakes Ring-billed and Herring Gulls. J. Field Ornithol., 67, 327–339.

Gaines, D. & Laymon, S.A. (1984). Decline, status, and preservation of the Yellow-billed Cuckoo in California. Western Birds, 15, 49–80.

Garnett, T. (2008). Personal communication to BirdLife (in litt.).

Garoche, J. & Sohier, A. (2008). La biologie du Pipit Maritime Anthus petrosus (Montagu) en Bretagne: caractère philopatrique et approche sur la dispersion natale. Alauda, 76, 23–33.

Gauger, V.H. (1999). Black Noddy. In The Birds of North America Online (A. Poole ed.). Cornell Laboratory of Ornithology, Ithaca.

Gauthier, G. (1993). Bufflehead, Bucephala albeola. In A. Poole & F. Gill (Eds.), The Birds of North America (No. 67). Academy of Natural Sciences, Philadelphia, Pennsylvania, USA, and American Ornithologists' Union, Washington, D.C., USA.

Gehlbach, F.R. & Gehlbach, N.Y. (2000). Whiskered Screech-owl. In The Birds of North America Online (A. Poole ed.). Cornell Laboratory of Ornithology, Ithaca.

Giesen, K.M. & Braun, C.E. (1993). Natal dispersal and recruitment of juvenile White-tailed Ptarmigan in Colorado. J. Wildl. Manage., 57, 72–77.

Gilchrist, H.G. (2001). Glaucous Gull. In The Birds of North America Online (A. Poole ed.). Cornell Laboratory of Ornithology, Ithaca.

Gill, R.E., Tomkovich, P.S. & McCaffery, B.J. (2002). Rock Sandpiper. In The Birds of North America Online (A. Poole ed.). Cornell Laboratory of Ornithology, Ithaca.

Goodrich, L.J., Crocoll, S.C. & Senner, S.E. (1996). Broad-winged Hawk. In The Birds of North America Online (A. Poole ed.). Cornell Laboratory of Ornithology, Ithaca.

Gossett, D.N. (1993). Studies of Ferruginous Hawk biology: I. Recoveries of banded Ferruginous Hawks from presumed eastern and western subpopulations. II. Morphological and genetic differences of presumed subpopulations of Ferruginous Hawks. III. Sex determination of nestling Ferruginous Hawks. Master’s Thesis, Boise State University.

Gowaty, P.A. & Plissner, J.H. (1998). Eastern Bluebird. In The Birds of North America Online (A. Poole ed.). Cornell Laboratory of Ornithology, Ithaca.

Gratson, M.W. (1988). Spatial patterns, movements, and cover selection by Sharp-tailed Grouse. In Adaptive Strategies and Population Ecology of Northern Grouse (A.T. Bergerund & M.W. Gratson eds). University of Minnesota Press.

Gratto, C.L., Morrison, R.I.G. & Cooke, F. (1985). Philopatry, site tenacity and mate fidelity in the Semipalmated Sandpiper. Auk, 102, 16–24.

Greene, E., Davison, W. & Muehter, V.R. (1998). Steller's Jay. In The Birds of North America Online (A. Poole ed.). Cornell Laboratory of Ornithology, Ithaca.

Greenwood, P.J. & Harvey, P.J. (1977). Feeding strategies and dispersal of territorial passerines: a comparative study of the Blackbird (Turdus merula) and the Greenfinch (Charduelis chloris). Ibis, 119, 528–531.

Grzybowski, J.A. (1991). Survivorship, dispersal and population structure of Black-capped Vireos at the Kerr Wildlife Management Area, Texas. Texas Parks Wildlife Department.

Gutiérrez, R.J., Franklin, A.B., Lahaye, W., Meretsky, V.J. & Ward, J.P. (1985). Juvenile Spotted Owl dispersal in northwestern California: preliminary results. In Ecology and Management of the Spotted Owl in the Pacific Northwest (R.J. Gutiérrez & A.B. Carey eds), pp. 60–65. U.S. Forest Service General Technical Report PNW-GTR-185.

Haas, C.A. (1995). Dispersal and use of corridors by birds in wooded patches on an agricultural landscape. Conserv. Biol., 9, 845–854.

Haftorn, S. (1997). Natal dispersal and winter flock formation in the Willow Tit (Parus montanus). Fauna Norvegica Series C Cinclus, 20, 17–35.

Haig, S.M. & Oring, L.W. (1988). Distribution and dispersal in the Piping Plover. Auk, 105, 630–638.

Halftorn, S. (1971). Norges Fugler. Universitetsforlaget, Oslo.

Halliburton, R. & Mewaldt, L.R. (1976). Survival and mobility in a population of Pacific Coast Song Sparrows (Melospiza melodia gouldii). Condor, 78, 499–504.

Hallworth, M., Ueland, A., Lambert, J.D. & Reitsma, L. (2008). Habitat selection and site fidelity of Canada Warblers (Wilsonia canadensis) in central New Hampshire. Auk, 125, 880–888.

Handbook of the Birds of the World (HBW) & Cornell Lab of Ornithology. (n.d.). Birds of the World. Cornell Lab of Ornithology. Available at: https://birdsoftheworld.org.

Handel, C.M. & Gill, R.E. Jr. (2000). Mate fidelity and breeding site tenacity in a monogamous sandpiper, the Black Turnstone. Anim. Behav., 60, 471–481.

Hanners, L.A. & Patton, S.R. (1998). Worm-eating Warbler. In The Birds of North America Online (A. Poole ed.). Cornell Laboratory of Ornithology, Ithaca.

Harmata, A.R., Montopoli, G.J., Oakleaf, B., Harmata, P.J. & Restani, M. (1999). Movements and survival of Bald Eagles banded in the Greater Yellowstone Ecosystem. J. Wildl. Manage., 63, 781–793.

Harrap, S. (2008). Family Certhiidae (Treecreepers). In Handbook of the Birds of the World Volume 13: Penduline-tits to Shrikes (J. del Hoyo, A. Elliott & D.A. Christie eds). Lynx Edicions, Barcelona.

Harris, M.P. (1983). Biology and survival of the immature Puffin, Fratercula arctica. Ibis, 125, 56–73.

Helm, B., Fiedler, W. & Callion, J. (2006). Movements of European Stonechats (Saxicola torquata) according to ringing discoveries. Ardea, 94, 33–44.

Hepp, G.R. & Bellrose, F.C. (1995). Wood Duck, Aix sponsa. In The Birds of North America (A. Poole & F. Gill eds), No. 169. Academy of Natural Sciences, Philadelphia, Pennsylvania, USA, and American Ornithologists' Union, Washington, D.C., USA.

Hines, J.E. (1986). Survival and reproduction of dispersing Blue Grouse. Condor, 88, 43–49.

Holmes, R.T., Sherry, T.W., Marra, P.P. & Petit, K.E. (1992). Multiple-brooding, nesting success, and annual productivity of a Neotropical migrant, the Black-throated Blue Warbler (Dendroica caerulescens), in an unfragmented temperate forest. Auk, 109, 321–333.

Hopp, S.L., Kirby, A. & Boone, C.A. (1995). White-eyed Vireo, Vireo griseus. In The Birds of North America (A. Poole & F. Gill eds), No. 168. Academy of Natural Sciences, Philadelphia, Pennsylvania, USA, and American Ornithologists' Union, Washington, D.C., USA.

Hunt, G.R. (1996). Family Rhynochetidae (Kagu). In Handbook of the Birds of the World Volume 3: Hoatzin to Auks (J. del Hoyo, A. Elliott & J. Sargatal eds). Lynx Edicions, Barcelona.

Islam, K. (1999). Erckel's Francolin (Francolinus erckelii), Black Francolin (Francolinus francolinus), and Gray Francolin (Francolinus pondicerianus). In The Birds of North America (A. Poole & F. Gill eds), No. 394-396. The Birds of North America, Inc., Philadelphia, PA.

Jackson, J.A. (1994). Red-cockaded Woodpecker, Picoides borealis. In The Birds of North America (A. Poole & F. Gill eds), No. 85. Academy of Natural Sciences, Philadelphia, Pennsylvania, USA, and American Ornithologists' Union, Washington, D.C., USA.

James, R.A. & Krementz, D.G. (2005). Dispersal patterns of Giant Canada Geese in the central United States. Proc. Southeast. Assoc. Fish Wildl. Agencies, 59, 144–154.

Jamieson, I.G. & Zwickel, F.C. (1983). Dispersal and site fidelity in Blue Grouse. Can. J. Zool., 61, 570–573.

Jehl, J.R. Jr. (1973). Breeding biology and systematic relationships of the Stilt Sandpiper. Wilson Bull., 85, 114–147.

Jenkins, D.G., Brescacin, C.R., Duxbury, C.V., Elliott, J.A., Evans, J.A., Grablow, K.R., et al. (2007). Does size matter for dispersal distance? Global Ecol. Biogeogr., 16, 415–425.

Johnson, O.W. & Connors, P.G. (2010). American Golden-Plover. In The Birds of North America Online (A. Poole ed.). Cornell Laboratory of Ornithology, Ithaca.

Johnson, R.F. (1956). Population structure in salt marsh song sparrows. Part I. Environment and annual cycle. Condor, 58, 24–44.

Keddy-Hector, D.P. (2000). Aplomado Falcon. In The Birds of North America Online (A. Poole ed.). Cornell Laboratory of Ornithology, Ithaca.

Keyser, A.J., Keyser, M.T. & Promislow, D.E.L. (2004). Life-history variation and demography in Western Bluebirds (Sialia mexicana) in Oregon. Auk, 121, 118–133.

Kim, S.-Y., Torres, R., Dominguez, C. & Drummond, H. (2007). Lifetime philopatry in the Blue-footed Booby: A longitudinal study. Behav. Ecol., 18, 1132–1138.

Kirk, D.A. & Mossman, M.J. (1998). Turkey Vulture. In The Birds of North America Online (A. Poole ed.). Cornell Laboratory of Ornithology, Ithaca.

Kirwan, G.M. (1996). Family Rostratulidae (Painted-snipes). In Handbook of the Birds of the World Volume 3: Hoatzin to Auks (J. del Hoyo, A. Elliott & J. Sargatal eds), pp. 374–396. Lynx Edicions, Barcelona.

Kochert, M.N., Steenhof, K., McIntyre, C.L. & Craig, E.H. (2002). Golden Eagle. In The Birds of North America Online (A. Poole ed.). Cornell Laboratory of Ornithology, Ithaca.

Korpimäki, E. (1987). Selection for nest-hole shift and tactics of breeding dispersal in Tengmalm's Owl (Aegolius funereus). J. Anim. Ecol., 56, 185–196.

Kroodsma, D.E. & Brewer, D. (2005). Family Troglodytidae (Wrens). In Handbook of the Birds of the World Volume 10: Cuckoo-shrikes to Thrushes (J. del Hoyo, A. Elliott & D.A. Christie eds), pp. 260–284. Lynx Edicions, Barcelona.

Kushlan, J.A. & Bildstein, K.L. (1992). White Ibis (Eudocimus albus). In The Birds of North America (A. Poole & F. Gill eds), No. 9. Academy of Natural Sciences, Philadelphia, Pennsylvania, USA, and American Ornithologists' Union, Washington, D.C.

Lanyon, W.E. (1995). Eastern Meadowlark. In The Birds of North America Online (A. Poole ed.). Cornell Laboratory of Ornithology, Ithaca.

Laskey, A.R. (1944). A study of the Cardinal in Tennessee. Wilson Bull., 56, 27–44.

Lavers, J., Hipfner, H., Chapdelaine, G. & Hipfner, J.M. (2009). Razorbill. In The Birds of North America Online (A. Poole ed.). Cornell Laboratory of Ornithology, Ithaca.

Lejeune, L., van de Pol, M., Cockburn, A., Louter, M., Brouwer, L. (2016). Male and female helper effects on maternal investment and adult survival in Red-winged Fairy-wrens. Behav. Ecol., 27, 1841–1850.

Lehman, V.W. (1984). Bobwhites in the Rio Grande Plain of Texas. Texas A&M University Press.

Lennington, S. & Mace, T. (1975). Mate fidelity and nesting site tenacity in the Killdeer. Auk, 92, 149–151.

Lens, L. & Matheve, H. (2009). Personal communication to BirdLife International (in litt.).

Lepson, J.K. & Freed, L.A. (1997). Akepa. In The Birds of North America Online (A. Poole ed.). Cornell Laboratory of Ornithology, Ithaca.

Lessells, C.M. (1985). Natal and breeding dispersal of wintering goshawks in Sweden. Viltrevy, 12, 1–36.

Lindberg, M.S. & Sedinger, J.S. (1997). Ecological consequences of nest site fidelity in Black Brant. Condor, 99, 25–38.

Lislevand, T., Byrkjedal, I. & Grønstøl, G.B. (2009). Dispersal and age at first breeding in Norwegian Northern Lapwings (Vanellus vanellus). Ornis Fennica, 86, 11–17.

Longcore, J.R., McAuley, D.G., Hepp, G.R. & Rhymer, J.M. (2000). American Black Duck. In The Birds of North America Online (A. Poole ed.). Cornell Laboratory of Ornithology, Ithaca.

Lowther, P.E. (1993). Brown-headed Cowbird (Molothrus ater). In The Birds of North America (A. Poole & F. Gill eds), No. 47. Academy of Natural Sciences, Philadelphia, Pennsylvania, USA, and American Ornithologists' Union, Washington, D.C.

Lowther, P.E., Celada, C., Klein, N.K., Rimmer, C.C. & Spector, D.A. (1999). Yellow Warbler. In The Birds of North America Online (A. Poole ed.). Cornell Laboratory of Ornithology, Ithaca.

Mallory, M. & Metz, K. (1999). Common Merganser. In The Birds of North America Online (A. Poole ed.). Cornell Laboratory of Ornithology, Ithaca.

Marchant, S., Higgins, P.J., Davies, S.J.J.F., Peter, J.M., Steele, W.K. & Cowling, S.J. (1990). Handbook of Australian, New Zealand and Antarctic Birds. Oxford University Press.

Marks, J.S. & Redmond, R.L. (1996). Demography of Bristle-thighed Curlews (Numenius tahitiensis) wintering on Laysan Island. Ibis, 138, 438–447.

Marti, C.D. (1999). Natal and breeding dispersal in Barn Owls. J. Raptor Res., 33, 181–189.

Martín, C.A., Alonso, J.C., Alonso, J.A., Palacín, C., Magaña, M. & Martín, B. (2008). Natal dispersal in Great Bustards: The effect of sex, local population size and spatial isolation. J. Anim. Ecol. 77, 326–334.

Martin, K. & Hannon, S.J. (1987). Natal philopatry and recruitment of Willow Ptarmigan in north central and northwestern Canada. Oecologia, 71, 518–524.

Martínez-Vilalta, A. & Motis, A. (1992). Family Ardeidae (Herons). In Handbook of the Birds of the World Volume 1: Ostrich to Ducks (J. del Hoyo, A. Elliott & J. Sargatal eds), pp. 272–312. Lynx Edicions, Barcelona.

Marzluff, J.M. & Balda, R.P. (1989). Causes and consequences of female-biased dispersal in a flock-living bird, the Pinyon Jay. Ecology, 70, 316–328.

Matthysen, E. & Schmidt, K.-H. (1987). Natal dispersal in the Nuthatch. Ornis Scandinavica, 18, 313–316.

Mazur, K.M. & James, P.C. (2000). Barred Owl. In The Birds of North America Online (A. Poole ed.). Cornell Laboratory of Ornithology, Ithaca.

McCamant, R.E. (1976). Nesting-box usage and mate loss behavior in the Black-bellied Whistling-Duck (Dendrocygna autumnalis). M.S. Thesis, Corpus Christi St. Univ., Corpus Christi. 72pp.

McCaskie, G. (1994). Flycatchers to Vireos. Southern Pac. Coast Reg.: Winter 1994 Field Notes, 46, 989.

McDonald, M.V. (1998). Kentucky Warbler. In The Birds of North America Online (A. Poole ed.). Cornell Laboratory of Ornithology, Ithaca.

McIntyre, C.L., Douglas, D.C. & Adams, L.G. (2009). Movements of juvenile Gyrfalcons from western and interior Alaska following departure from their natal areas. J. Raptor Res., 43, 99–109.

Mead, C.J. (1979). Colony fidelity and interchange in the Sand Martin. Bird Study, 26, 99–106.

Mearns, R. & Newton, I. (1982). Turnover and dispersal in a Peregrine (Falco peregrinus) population. Ibis, 126, 347–355.

Melling, T., Dudley, S. & Doherty, P. (2011). Status and movements of Eagle Owls in Europe. Brit. Birds, 104, 544–546.

Melvin, S.L., Gawlik, D.E. & Scharff, T. (1999). Long-term movement patterns for seven species of wading birds. Waterbirds, 22, 411–416.

Mennill, D.J. & Rogers, A.C. (2006). Whip it good! Geographic consistency in male songs and variability in female songs of the duetting Eastern Whipbird (Psophodes olivaceus). J. Avian Biol., 37, 93–100.

Middleton, A.L.A. (1979). Influence of age and habitat on reproduction by the American Goldfinch. Ecology, 60, 418–432.

Miller, G.S. & Meslow, E.C. (1985). Dispersal data for juvenile Spotted Owls: The problem of small sample size. In Ecology and management of the Spotted Owl in the Pacific Northwest (R.J. Gutiérrez & A.B. Carey eds), pp. 69–73. U.S. Forest Service General Technical Report GTR PNW-185.

Miller, K.E. & Smallwood, J.A. (1997). Natal dispersal and philopatry of southeastern American Kestrels in Florida. Wilson Bull., 109, 226–232.

Molina, K.C., Garrett, K.L. & Larson, K.W. (2009). The winter distribution of the Western Gull-billed Tern (Gelochelidon nilotica vanrossemi). Western Birds, 40, 2–20.

Monaghan, P. (2002). Arctic Tern. In The Migration Atlas: Movements of the Birds of Britain and Ireland (C.V. Wernham, M.P. Toms, J.H. Marchant, J.A. Clark, G.M. Siriwardena & S.R. Baillie eds), pp. 52–53. T. and A. D. Poyser, London.

Montgomerie, R. & Lyon, B. (2011). Snow Bunting. In The Birds of North America Online (A. Poole ed.). Cornell Laboratory of Ornithology, Ithaca.

Moore, W.S. & Dolbeer, R.A. (1989). The use of banding recovery data to estimate dispersal rates and gene flow in avian species: Case studies in the Red-winged Blackbird and Common Grackle. Condor, 91, 242–253.

Mountjoy, D.J. (2005). Family Bombycillidae (Waxwings). In Handbook of the Birds of the World Volume 10: Cuckoo-shrikes to Thrushes (J. del Hoyo, A. Elliott & D.A. Christie eds), pp. 499–527. Lynx Edicions, Barcelona.

Mulder, R.A. (1995). Natal and breeding dispersal in a cooperative, extra-group-mating bird. J. Avian Biol., 26, 234–240.

Negro, J.J., Hiraldo, F. & Donázar, J.A. (1997). Causes of natal dispersal in the Lesser Kestrel: Inbreeding avoidance or resource competition? J. Anim. Ecol., 66, 640–648.

Nelson, S.K. (1997). Marbled Murrelet. In The Birds of North America Online (A. Poole ed.). Cornell Laboratory of Ornithology, Ithaca.

Nemeth, N.M. & Morrison, J.L. (2002). Natal dispersal of the Crested Caracara (Caracara cheriway) in Florida. J. Raptor Res., 36, 203–206.

Nice, M.M. (1937). Studies in the life history of the Song Sparrow. I. A population study of the Song Sparrow. Trans. Linnean Soc. New York, 6, 1–328.

Nilsson, J.A. (1989). Causes and consequences of natal dispersal in the Marsh Tit (Parus palustris). J. Anim. Ecol., 58, 619–636.

Nolan, V.Jr. (1978). The Ecology and Behavior of the Prairie Warbler (Dendroica discolor). Ornithological Monographs, 26, 1–595.

Norris, R.A. (1958). Comparative biosystematics and life history of the Nuthatches Sitta pygmaea and Sitta pusilla. Univ. Calif. Publ. Zool., 56, 119–300.

Okill, J.D. (1992). Natal dispersal and breeding site fidelity of Red-throated Divers (Gavia stellata) in Shetland. Ringing Migr., 13, 57–58.

O'Neill, P., Heatwole, H., Preker, M. & Jones, M. (1996). Populations, movements and site fidelity of Brown and Masked Boobies on the Swain Reefs, Great Barrier Reef, as shown by banding recoveries. CRC Reef Research Centre, Townsville.

Oring, L.W., Gray, E.M. & Reed, J.M. (1997). Spotted Sandpiper (Actitis macularia). In The Birds of North America (A. Poole & F. Gill eds), No. 289. Academy of Natural Sciences, Philadelphia, Pennsylvania, USA, and American Ornithologists' Union, Washington, D.C., USA.

Orta, J. (1992). Family Fregatidae (Frigatebirds). In Handbook of the Birds of the World Volume 1: Ostrich to Ducks (J. del Hoyo, A. Elliott & J. Sargatal eds), pp. 375–399. Lynx Edicions, Barcelona.

Paradis, E., Baillie, S.R., Sutherland, W.J. & Gregory, R.D. (1998). Patterns of natal and breeding dispersal in birds. J. Anim. Ecol., 67, 518–536.

Parmelee, D.F., Stephens, H.A. & Schmidt, R.H. (1967). The Birds of Southeastern Victoria Island and Adjacent Small Islands. National Museum of Canada Bulletin, 222, 1–229.

Pärt, T. (1990). Natal dispersal in the Collared Flycatcher: Possible causes and reproductive consequences. Ornis Scandinavica, 21, 83–88.

Payne, R.B. & Payne, L.L. (1993). Breeding dispersal in Indigo Buntings: Circumstances and consequences for breeding success and population structure. Condor, 95, 1–24.

Peterson, M.J. (2000). Plain Chachalaca (Ortalis vetula), version 2.0. In The Birds of North America (A.F. Poole & F.B. Gill eds). Cornell Lab of Ornithology, Ithaca, NY, USA.

Peterson, M.R., Grand, B.J. & Dau, C.P. (2000). Spectacled Eider. In The Birds of North America Online (A. Poole ed.). Cornell Laboratory of Ornithology, Ithaca.

Poole, A.F. (1994). Family Pandionidae (Osprey). In Handbook of the Birds of the World Volume 2: New World Vultures to Guineafowl (J. del Hoyo, A. Elliott & J. Sargatal eds), pp. 484–504. Lynx Edicions, Barcelona.

Poole, A.F., Bevier, L.R., Marantz, C.A. & Meanley, B. (2005). King Rail. In The Birds of North America Online (A. Poole ed.). Cornell Laboratory of Ornithology, Ithaca.

Post, W., Poston, J.P. & Bancroft, G.T. (1996). Boat-tailed Grackle (Quiscalus major). In The Birds of North America (A. Poole & F. Gill eds), No. 207. Academy of Natural Sciences, Philadelphia, Pennsylvania, USA, and American Ornithologists' Union, Washington, D.C., USA.

Powers, D.R. & Wethington, S.M. (1999). Broad-billed Hummingbird. In The Birds of North America Online (A. Poole ed.). Cornell Laboratory of Ornithology, Ithaca.

Pratt, T.K., Banko, P.C., Fancy, S.G., Lindsey, G.D. & Jacobi, J.D. (1997). Status and management of the Palila, an endangered honeycreeper, 1987–1996. Pac. Conserv., 3, 330–340.

Preston, C.R. & Beane, R.D. (1993). Red-tailed Hawk (Buteo jamaicensis). In The Birds of North America (A. Poole & F. Gill eds), No. 52. Academy of Natural Sciences, Philadelphia, Pennsylvania, USA, and American Ornithologists' Union, Washington, D.C., USA.

Price, J.B. (1936). The family relations of the Plain Titmouse. Condor, 38, 23–28.

Proudfoot, G.A. & Johnson, R.R. (2000). Ferruginous Pygmy-Owl (Glaucidium brasilianum). In The Birds of North America (A. Poole & F. Gill eds), No. 498. The Birds of North America, Inc., Philadelphia, PA, USA.

Raine, A.F., Sowter, D.J., Brown, A.F. & Sutherland, W.J. (2006). Natal philopatry and local movement patterns of Twite (Carduelis flavirostris). Ringing Migr., 23, 89–94.

Regosin, J.V. & Pruett-Jones, S. (1995). Aspects of breeding biology and social organization in the Scissor-tailed Flycatcher. Condor, 97, 154–164.

Reynolds, T.D., Rich, T.D. & Stephens, D.A. (1999). Sage Thrasher. In The Birds of North America Online (A. Poole ed.). Cornell Laboratory of Ornithology, Ithaca.

Rheinwald, G. (1975). The pattern of settling distances in a population of House Martins (Delichon urbica). Ardea, 63, 136–145.

Rimmer, C.C., McFarland, K.P., Ellison, W.G. & Goetz, J.E. (2001). Bicknell's Thrush. In The Birds of North America Online (A. Poole ed.). Cornell Laboratory of Ornithology, Ithaca.

Robert, M. & Laporte, P. (1993). Le Râle Jaune (Coturnicops noveboracensis) à l'Ile aux Grues et à Cacouna. Service Canadien de la Faune, Sainte-Foy.

Robertson, G.J. & Gouldie, R.I. (1999). Harlequin Duck. In The Birds of North America Online (A. Poole ed.). Cornell Laboratory of Ornithology, Ithaca.

Robertson, W.B., Breen, L.L. & Patty, B.W. (1983). Movement of marked Roseate Spoonbills in Florida with a review of present distribution. J. Field Ornithol., 54, 225–236.

Robinson, J.A. & Oring, L.W. (1997). Natal and breeding dispersal in American Avocets. Auk, 114, 416–430.

Robinson, W.D. (1995). Louisiana Waterthrush (Seiurus motacilla). In The Birds of North America (A. Poole & F. Gill eds), No. 151. Academy of Natural Sciences, Philadelphia, Pennsylvania, USA, and American Ornithologists' Union, Washington, D.C., USA.

Rodewald, P.G., Withgott, J.H. & Smith, K.G. (1999). Pine Warbler. In The Birds of North America Online (A. Poole ed.). Cornell Laboratory of Ornithology, Ithaca.

Rosenfield, R.N. & Bielefeldt, J. (1992). Natal dispersal and inbreeding in the Cooper's Hawk. Wilson Bull., 104, 182–184.

Russell, E.M. & Rowley, I. (1993). Philopatry or dispersal: Competition for territory vacancies in the splendid fairy-wren (Malurus splendens). Anim. Behav., 45, 519–539.

Ryder, R.A. (1972). Avian population studies on the Pawnee site, 1968–1971. United States International Biological Program.

Šálek, M. & Marhoul, P. (2008). Spatial movements of Grey Partridge (Perdix perdix): Male-biased spring dispersal and effect of habitat quality. J. Ornithol., 149, 329–335.

Sankamethawee, W., Hardesty, B.D. & Gale, G.A. (2010). Sex-bias and timing of natal dispersal in cooperatively breeding Puff-throated Bulbuls (Alophoixus pallidus). J. Ornithol., 151, 779–789.

Sawaya, P.L. (1990). A detailed analysis of the genetic interaction at a hybrid zone between the Chickadees Parus atricapillus and P. carolinensis as revealed by nuclear and mitochondrial DNA restriction fragment length variation. PhD Thesis, University of Cincinnati.

Schreiber, E.A. (2003). Breeding biology and ecology of the seabirds of Johnson Atoll, Central Pacific Ocean, 2003: Long-term monitoring for effects of Johnson Atoll Chemical Demilitarisation Project 1984–2003. Department of Defense, Aberdeen.

Schroeder, M.A. & Braun, C.E. (1993). Movement and philopatry of Band-tailed Pigeons captured in Colorado. J. Wildl. Manag., 57, 103–112.

Scofield, R.P. (2009). Family Callaeidae (New Zealand Wattlebirds). In Handbook of the Birds of the World Volume 14: Bush-shrikes to Old World Sparrows (J. del Hoyo, A. Elliott & D.A. Christie eds), pp. 291–310. Lynx Edicions, Barcelona.

Scott, L. & Lane, J. (1974). Mountain Bluebird travels 130 miles to renest. Blue Jay, 32, 44–45.

Seaton, R., Holland, J.D., Minot, E.O. & Springett, B.P. (2008). Natal dispersal of New Zealand Falcon (Falco novaeseelandiae) in plantation forests. Notornis, 55, 140–145.

Sekercioglu, C.H., Loarie, S.R., Oviedo Brenes, F., Ehrlich, P.R. & Daily, G.C. (2007). Persistence of forest birds in the Costa Rican agricultural countryside. Conserv. Biol., 21, 482–494.

Sheldon, W.G. (1953). Woodcock studies in Massachusetts. Trans. North Am. Wildl. Nat. Resour. Conf., 18, 369–377.

Sherry, T.W. & Holmes, R.T. (1997). American Redstart. In The Birds of North America Online (A. Poole ed.). Cornell Laboratory of Ornithology, Ithaca.

Shields, W.M. (1982). Optimal inbreeding and the evolution of philopatry. In The Ecology of Animal Movement (I.R. Swingland & P.J. Greenwood eds), pp. 132–159. Oxford University Press, Oxford, UK.

Sick, H. (1993). Birds in Brazil: A Natural History. Princeton University Press.

Skeel, M.A. & Mallory, E. (1996). Whimbrel (Numenius phaeopus). In The Birds of North America (A. Poole & F. Gill eds), No. 219. Academy of Natural Sciences, Philadelphia, Pennsylvania, USA, and American Ornithologists' Union, Washington, D.C., USA.

Snetsinger, T.J., Wakely, K.M. & Fancy, S.G. (1999). Puaiohi. In The Birds of North America Online (A. Poole ed.). Cornell Laboratory of Ornithology, Ithaca.

Snyder, N.R.F., Ramey, R.R. & Sibley, F.C. (1986). Nest-site biology of the California Condor. Condor, 88, 228–241.

Soikkeli, M. (1970). Dispersal of Dunlin (Calidris alpina) in relation to sites of birth and breeding. Ornis Fennica, 47, 1–9.

Southern, H.N. (1970). The natural control of a population of tawny owls (Strix aluco). J. Zool. Lond., 162, 197–285.

Spear, L.B., Pyle, P. & Nur, N. (1998) Natal dispersal in the Western Gull: Proximal factors and fitness consequences. Journal of Animal Ecology, 67, 165–179.

Stacier, C.A. (1992) Social behavior of the Northern Parula, Cape May Warbler, and Prairie Warbler wintering in second-growth forest in southwestern Puerto Rico. In Ecology and Conservation of Neotropical Migrant Landbirds (J.M. Hagan & D.W. Johnston, eds). Smithsonian Institution Press, Washington, D.C.

Steeger, C. & Dulisse, J. (1997) Ecological interrelationships of Three-toed Woodpeckers with bark beetles and pine trees. Forest Sciences, Nelson Forest Region.

Steenhof, K. (1998) Prairie Falcon (Falco mexicanus). In The Birds of North America, No. 346 (A. Poole & F. Gill, eds.). The Birds of North America, Inc., Philadelphia, PA.

Stenzel, L.E., Page, G.W., Warriner, J.C., Warriner, J.S., George, D.E., Eyster, C.R., Ramer, B.A. & Neuman, K.K. (2007) Survival and natal dispersal of juvenile Snowy Plovers (Charadrius alexandrinus) in central coastal California. Auk, 124, 1023–1036.

Strickland, D. (1991) Juvenile dispersal in gray jays: Dominant brood member expels siblings from natal territory. Canadian Journal of Zoology, 69, 2935–2945.

Szabo, J. (2011). Personal communication to BirdLife International (in litt.).

Tarvin, K.A. (1998) The influence of habitat variation on demography of Blue Jays (Cyanocitta cristata) in south-central Florida. PhD Thesis, University of South Florida.

Tate, G.R. (1992) Short-eared Owl (Asio flammeus). In Migratory Nongame Birds of Management Concern in the Northeast (K.J. Schneider & D.M. Pence, eds). United States Fish and Wildlife Service, Newton Corner.

Telfair, R.C., II & Morrison, M.L. (1995) Neotropic Cormorant (Phalacrocorax brasilianus). In The Birds of North America, No. 137 (A. Poole & F. Gill, eds). Academy of Natural Sciences, Philadelphia, PA & American Ornithologists' Union, Washington, DC, USA.

Tenney, C.R. (2000) Northern Beardless-Tyrannulet. In The Birds of North America Online (A. Poole, ed.). Cornell Laboratory of Ornithology, Ithaca.

Thomas, B.T. (1996) Family Opisthocomidae (Hoatzin). In Handbook of the Birds of the World Volume 3: Hoatzin to Auks (J. del Hoyo, A. Elliott & J. Sargatal, eds). Lynx Edicions, Barcelona.

Tobias, J. (unpublished data), in Stewart, P.S., Voskamp, A., Santini, L., Biber, M.F., Devenish, A.J.M., Hof, C., et al. (2022). Global impacts of climate change on avian functional diversity. Ecology Letters, 25, 673–685.

Tomlinson, R.E., Wight, H.M. & Baskett, T.S. (1960) Migrational homing, local movement, and mortality of mourning doves in Missouri. In Transactions of the 25th North American Wildlife Conference (Dallas, Texas, USA, 1960). Wildlife Management Institute, Washington, D.C., USA.

Turner, A.K. (2004) Family Hirundinidae (Swallows and Martins). In Handbook of the Birds of the World Volume 9: Cotingas to Pipits and Wagtails (J. del Hoyo, A. Elliott & D.A. Christie, eds). Lynx Edicions, Barcelona.

Tweit, R.C. (1996) Curve-billed Thrasher, Toxostoma curvirostre. In The Birds of North America No. 235 (A. Poole & F. Gill, eds). Academy of Natural Sciences, Philadelphia, PA & American Ornithologists' Union, Washington, D.C., USA.

Van Balen, S. (2008) Family Zosteropidae (White-eyes). In Handbook of the Birds of the World Volume 13: Penduline-tits to Shrikes (J. del Hoyo, A. Elliott & D.A. Christie, eds). Lynx Edicions, Barcelona.

Van Houtan, K.S., Pimm, S.L., Halley, J.M., Bierregaard, R.O., Jr & Lovejoy, T.E. (2007) Dispersal of Amazonian birds in continuous and fragmented forest. Ecology Letters, 10, 219–229.

Vandenbulcke, P. (1989) Origin, primary moult and biometry of the Great Black-backed Gull, Larus marinus, on the Belgian coast. Gerfaut, 79, 31–53.

Verbeek, N.A. & Butler, R.W. (1999) Northwestern Crow. In The Birds of North America Online (A. Poole, ed.). Cornell Laboratory of Ornithology, Ithaca.

Verner, J. (1971) Survival and dispersal of male Long-billed Marsh Wrens. Bird Banding, 42, 92–98.

Wallin, K. & Andersson, M. (1981) Adult nomadism in Tengmalm's owl Aegolius funereus. Ornis Scandinavica, 12, 125–126.

Weise, C.M. & Meyer, J.R. (1979) Juvenile dispersal and development of site-fidelity in the Black-capped Chickadee. The Auk, 96, 40–55.

West, S. (1995) Cave Swallow, Hirundo fulva. In The Birds of North America (A. Poole & F. Gill, eds). Academy of Natural Sciences, Philadelphia, PA & American Ornithologists' Union, Washington, D.C., USA.

Whitfield, D.P., Duffy, K., McLeod, D.R.A., Evans, R.J., Maclennan, A.M., Reid, R., Sexton, D., Wilson, J.D. & Douse, A. (2009) Juvenile dispersal of White-tailed Eagles in Western Scotland. Journal of Raptor Research, 43, 110–120.

Wiens, J.D., Reynolds, R.T. & Noon, B.R. (2006) Juvenile movement and natal dispersal of Northern Goshawks in Arizona. The Condor, 108, 253–269.

Winkler, H. & Christie, D.A. (2002) Family Picidae (Woodpeckers). In Handbook of the Birds of the World Volume 7: Jacamars to Woodpeckers (J. del Hoyo, A. Elliott & J. Sargatal, eds). Lynx Edicions, Barcelona.

Withey, J.C. & Marzluff, J.M. (2005) Dispersal by juvenile American Crows (Corvus brachyrhynchos) influences population dynamics across a gradient of urbanization. The Auk, 122(1), 205–221.

Wolf, B.O. (1997) Black Phoebe, Sayornis nigricans. In The Birds of North America (A. Poole & F. Gill, eds). Academy of Natural Sciences, Philadelphia, PA & American Ornithologists' Union, Washington, D.C., USA.

Woltmann, S., Terrill, R.S., Miller, M.J. & Brady, M.L. (2010) Chestnut-backed Antbird (Myrmeciza exsul). In Neotropical Birds Online (T.S. Schulenberg, ed.). Cornell Laboratory of Ornithology, Ithaca.

Wood, D.A., Gore, J.A., Nesbitt, S.A. & Sasser, M.S. (1995) Dispersal of Brown Pelicans from a natal site in Bay County, Florida, with an update on Brown Pelican status in Florida. Proceedings of the Southeastern Association of Fish and Wildlife Agencies, 49, 367–371.

Woodall, P.F. (2001) Family Alcedinidae (Kingfishers). In Handbook of the Birds of the World Volume 6: Mousebirds to Hornbills (J. del Hoyo, A. Elliott & J. Sargatal, eds). Lynx Edicions, Barcelona.

Woodworth, B.L., Nelson, J.T., Tweed, E.J., Fancy, S.G., Moore, M.P., Cohen, E.B. & Collins, M.S. (2001) Breeding productivity and survival of the endangered Hawai'i Creeper in a wet forest refuge on Mauna Kea, Hawai'i. Studies in Avian Biology, 22, 164–172.

Woolfenden, G.E. & Fitzpatrick, J.W. (1978) The inheritance of territory in group-breeding birds. BioScience, 28, 104–108.

Wright, A.L., Hayward, G.D., Matsouka, S.M. & Hayward, P.H. (1998) Townsend's Warbler. In The Birds of North America Online (A. Poole, ed.). Cornell Laboratory of Ornithology, Ithaca.

Wyatt, V.E. & Francis, C.M. (2002) Rose-breasted Grosbeak. In The Birds of North America Online (A. Poole, ed.). Cornell Laboratory of Ornithology, Ithaca.

Yerkes, T. (2000) Influence of female age and body mass on brood and duckling survival, number of surviving ducklings, and brood movements in Redheads. Condor, 102, 926–929.

Zembal, R., Fancher, J.M., Nordby, C.S. & Bransfield, R.J. (1985) Intermarsh movements by Light-footed Clapper Rails indicated in part through regular censusing. California Department of Fish and Game.

**Running mammal:**

Aars, J., Lambin, X., Denny, R. & Griffin, A.C. (2001) Water vole in the Scottish uplands: distribution patterns of disturbed and pristine populations ahead and behind the American mink invasion front. Animal Conservation, 4, 187–194.

Ables, E. (1965) An exceptional fox movement. Journal of Mammalogy, 46, 102.

Allen, D.L. (1939) Michigan cottontails in winter. Journal of Wildlife Management, 3, 307–322.

Allen, S.H. & Sargent, A. B. (1993) Dispersal patterns of red foxes relative to population density. Journal of Wildlife Management, 57(3), 526.

Allred, D.M. & Beck, D.E. (1963) Range of movement and dispersal of some rodents at the Nevada atomic test site. Journal of Mammalogy, 44, 190–200.

Andrews, R.D. & Boggess, E.K. (1978) Ecology of coyotes in Iowa. In M. Bekoff (Ed.), Coyotes: Biology, Behavior, and Management (pp. 249–265). Academic Press, New York, New York, USA.

Andrews, R.D. & Bekoff, M. (1978) Coyotes: Biology, Behavior, and Management. Academic Press.

Angerbjörn, A. & Flux, J.E.C. (1995) Lepus timidus. Mammalian Species, 495, 1–11.

Angerbjörn, A., Hersteinsson, P. & Tannerfeldt, M. (2004) Arctic foxes. In D. W. Macdonald & C. Sillero-Zubiri (Eds.), The Biology and Conservation of Canids (pp. 163–172). Oxford University Press, Oxford.

Arjo, W.M., Huenefeld, R.E. & Nolte, D.L. (2007) Mountain beaver home ranges, habitat use, and population dynamics in Washington. Canadian Journal of Zoology, 85, 328–333.

Armitage, K.B. (1991) Social and population dynamics of yellow-bellied marmots: Results from long-term research. Annual Review of Ecology and Systematics, 22, 379–407.

Armitage, K.B. & Downhower, J.F. (1974) Demography of yellow-bellied marmot populations. Ecology, 55, 1233–1245.

Arthur, S.M., Paragi, T.F., Krohn, W. B. (1993) Dispersal of juvenile fishers in Maine. Journal of Wildlife Management, 57(4), 868–874.

Aubry, K., Wisely, S., Raley, C. & Buskirk, S. (2005) Zoogeography, spacing patterns, and dispersal in fishers. In D.J. Harrison, A.K. Fuller & G. Proulx (Eds.), Martens and Fishers (Martes) in Human-Altered Environments (pp. 201–220). Springer, New York.

Avril, A., Léonard, Y., Letty, J., Péroux, R., Guitton, J.S. & Pontier, D. (2011) Natal dispersal of European hare in a high-density population. Mammalian Biology - Zeitschrift für Säugetierkunde, 76, 148–156.

Ballard, W.B., Whitman, J.S. & Gardner, C.L. (1987) Ecology of an exploited wolf population in south-central Alaska. Wildlife Monographs, 98, 3–54.

Begg, C.M., Begg, K.S., et al. (2005) Spatial organization of the honey badger, Mellivora capensis, in the southern Kalahari: home-range size and movement patterns. Journal of Zoology, 265, 23–35.

Beier, P. (1995) Dispersal of juvenile cougars in fragmented habitat. Journal of Wildlife Management, 59(2), 228–237.

Berg, W.E. & Kuehn, D.W. (1982) Ecology of wolves in north-central Minnesota. In F.C. Harrington & P.C. Paquet (Eds.), Wolves of the World: Perspectives of Behaviour, Ecology and Conservation (pp. 4–11). Noyes, Park Ridge, New Jersey, USA.

Berger, J. (1987) Reproductive fates of dispersers in a harem-dwelling ungulate: the wild horse. In B.D. Chepko-Sade & Z.T. Halpin (Eds.), Mammalian Dispersal Patterns: The Effects of Social Structure on Population Genetics (pp. 41–54). University of Chicago Press, Chicago.

Berry, R. (1968) The ecology of an island population of the house mouse. Journal of Animal Ecology, 37, 445–470.

Best, T.L. (1996) Lepus californicus. Mammalian Species, 530, 1–10.

Bjorge, R.R., Gunson, J.R. & Samuel, W.M. (1981) Population characteristics and movements of striped skunks (Mephitis mephitis) in central Alberta. Canadian Field-Naturalist, 95, 149–155.

Blanco, J.C. & Cortes, Y. (2007) Dispersal patterns, social structure and mortality of wolves living in agricultural habitats in Spain. Journal of Zoology, 273, 114–124.

Bothma, J.P. (1971) Notes on the movement by the black-backed jackal and the aardwolf in the western Transvaal. Zoologica Africana, 6, 205–207.

Bovet, J. (1980) Homing behavior and orientation in the red-backed vole, Clethrionomys gapperi. Canadian Journal of Zoology, 58, 754–760.

Bowen, W.D. (1982) Home range and spatial organization of coyotes in Jasper National Park, Alberta. Journal of Wildlife Management, 46, 201–216.

Bowers, G.L. (1954) An evaluation of cottontail rabbit management in Pennsylvania. In Transactions of the North American Wildlife Conference (pp. 358–367).

Bowman, J., Forbes, G. & Dilworth, T.G. (2000) Distances moved by small woodland rodents within large trapping grids. Canadian Field-Naturalist, 115, 64–67.

Boyce, C.C.K. & Boyce, J.L. III (1988) Population biology of Microtus arvalis. II. Natal and breeding dispersal of females. Journal of Animal Ecology, 57, 723–736.

Boyd, D.K. & Pletscher, D.H. (1999) Characteristics of dispersal in a colonizing wolf population in the Central Rocky Mountains. Journal of Wildlife Management, 63(4), 1094–1108.

Boyd, J.M. (1963) Home range and homing experiments with the St. Kilda field-mouse. Proceedings of the Zoological Society of London, 1–14.

Boyd, D.K., Paquet, P.C., Donelon, S., Ream, R.R., Pletscher, D.H., & White, C.C. (1995). Transboundary movements of a colonizing wolf population in the Rocky Mountains. In: Carbyn, L.N., Fritts, S.H., & Seip, D.R. (eds). Ecology and Conservation of Wolves in a Changing World, pp. 135–140. Canadian Circumpolar Institute, Edmonton, Alberta, Canada.

Boydston, E.E., Kapheim, K.M. & Holekamp, K.E. (2005) Sexually dimorphic patterns of space use throughout ontogeny in the spotted hyena (Crocuta crocuta). Journal of Zoology, 267, 271–281.

Bradley, A. (1997) Reproduction and life history in the red-tailed phascogale, Phascogale calura (Marsupialia: Dasyuridae): the adaptive-stress senescence hypothesis. Journal of Zoology, 241, 739–755.

Bray, Y., Devillard, S., et al. (2007) Natal dispersal of European hare in France. Journal of Zoology, 273, 426–434.

Brazda, A.R. (1953) Elk migration patterns, and some of the factors affecting movements in the Gallatin River drainage, Montana. Journal of Wildlife Management, 17, 9–23.

Broadbooks, H.E. (1961) Homing behavior of deer mice and pocket mice. Journal of Mammalogy, 42, 416–417.

Broadbooks, H.E. (1970) Home ranges and territorial behavior of the yellow-pine chipmunk, Eutamias amoenus. Journal of Mammalogy, 51, 310–326.

Broekhuizen, S. & Maaskamp, F. (1982) Movement, home range and clustering in the European hare (Lepus europaeus Pallas) in The Netherlands. Zeitschrift für Säugetierkunde, 47, 22–32.

Broquet, T., Johnson, C., Petit, E., Thompson, I., Burel, F. & Fryxell, J. (2006) Dispersal and genetic structure in the American marten, Martes americana. Molecular Ecology, 15, 1689–1697.

Büchner, S. (2008) Dispersal of common dormice Muscardinus avellanarius in a habitat mosaic. Acta Theriologica, 53, 259–262.

Bunnell, F.L. & Harestad, A.S. (1983) Dispersal and dispersion of black-tailed deer: models and observations. Journal of Mammalogy, 64, 201–209.

Burt, W.H. (1940) Territorial behavior and populations of some small mammals in southern Michigan. Miscellaneous Publications, Museum of Zoology, University of Michigan, 40, 1–32.

Byrom, A.E. & Krebs, C.J. (1999) Natal dispersal of juvenile arctic ground squirrels in the boreal forest. Canadian Journal of Zoology, 77, 1048–1059.

Carbyn, L.N., Armbruster, H.J., Mamo, C., Bowles, M.L. & Whelan, C.J. (1994) The swift fox reintroduction program in Canada from 1983 to 1992. In Restoration of Endangered Species—Conceptual Issues, Planning and Implementation (pp. 197–212).

Cederlund, G.N. & Sand, H.K.G. (1992) Dispersal of subadult moose (Alces alces) in a nonmigratory population. Canadian Journal of Zoology, 70, 1309–1314.

Chamberlain, P.A. (1980) Armadillos: problems and control. In Proceedings of the Vertebrate Pest Conference 9, 163–169.

Chapman, J.A. & Trethewey, D.E.C. (1972) Movements within a population of introduced eastern cottontail rabbits. Journal of Wildlife Management, 36, 155–158.

Cheesman, C.L., Creswell, W.J., Harris, S. & Mallinson, P.J. (1988) Comparison of dispersal and other movements in two badger (Meles meles) populations. Mammal Review, 18, 51–59.

Cheney, D.L. & Seyfarth, R.M. (1983) Nonrandom dispersal in free-ranging vervet monkeys: social and genetic consequences. The American Naturalist, 122, 392–412.

Ciucci, P., Reggioni, W., Maiorano, L. & Boitani, L. (2009) Long-distance dispersal of a rescued wolf from the Northern Apennines to the Western Alps. Journal of Wildlife Management, 73, 1300–1306.

Clark, T.W., Hoffmann, R.S. & Nadler, C.F. (1971) Cynomys leucurus. Mammalian Species, 7, 1–4.

Clark, W.R., Hasbrouck, J.J., Kienzler, J.M. & Glueck, T.F. (1989) Vital statistics and harvest of an Iowa raccoon population. Journal of Wildlife Management, 53, 982–990.

Clutton-Brock, T.H., Guinness, F.E. & Albon, S.D. (1982) Red Deer: Behavior and Ecology of Two Sexes. University of Chicago Press.

Costello, C.M. (2010) Estimates of dispersal and home-range fidelity in American black bears. Journal of Mammalogy, 91(1), 116–121.

Crockett, C.M. (1985) Population studies of red howler monkeys (Alouatta seniculus). National Geographic Research, 1, 264–273.

da Silva, K., Mahan, C. & da Silva, J. (2002) The trill of the chase: eastern chipmunks call to warn kin. Journal of Mammalogy, 83, 546–552.

Dahl, F. & Willebrand, T. (2005) Natal dispersal, adult home ranges and site fidelity of mountain hares (Lepus timidus) in the boreal forest of Sweden. Wildlife Biology, 11, 309–317.

Dalke, P.D. & Sime, P.R. (1938) Home and seasonal ranges of the eastern cottontail in Connecticut. In Transactions of the North American Wildlife Conference (pp. 659–669).

Daly, J.C. & Patton, J. (1990) Dispersal, gene flow, and allelic diversity between local populations of Thomomys bottae pocket gophers in the coastal ranges of California. Evolution, 44, 1283–1294.

Danner, D.A. & Fisher, A.R. (1977) Evidence of homing by a coyote (Canis latrans). Journal of Mammalogy, 58, 244–245.

de Vos, A. (1952) The ecology and management of fisher and marten in Ontario. Technical Bulletin, Ontario Department of Lands and Forests, 90.

DeBusk, J. & Kennerly Jr, T.E. (1975) Homing in the cotton rat, Sigmodon hispidus. American Midland Naturalist, 93, 149–157.

Dice, L.R. & Howard, W.E. (1951) Distance of dispersal by prairie deermice from birthplaces to breeding sites. Contributions from the Laboratory of Vertebrate Biology, 50, 1–13.

Dickman, C.R. & Doncaster, C.P. (1989) The ecology of small mammals in urban habitats. II. Demography and dispersal. Journal of Animal Ecology, 58, 119–127.

Doncaster, C.P., Rondinini, C. & Johnson, P.C. (2001) Field test for environmental correlates of dispersal in hedgehogs (Erinaceus europaeus). Journal of Animal Ecology, 70, 33–46.

Dusek, G., Mackie, R., Herriges Jr, J. & BB, C. (1989) Population ecology of white-tailed deer along the lower Yellowstone River. Wildlife Monographs, 104, 1–68.

Eberhardt, L.L. & Hanson, W.D. (1978) Long-distance movements of arctic foxes tagged in northern Alaska. Canadian Field-Naturalist, 92, 146–148.

Egoscue, H.J. (1956) Preliminary studies of the kit fox in Utah. Journal of Mammalogy, 37, 351–357.

Elbroch, M., Wittmer, H.U., Saucedo, C. & Corti, P. (2009) Long-distance dispersal of a male puma (Puma concolor puma) in Patagonia. Revista Chilena de Historia Natural, 82, 459–461.

Ellis, R.J. (1964) Tracking raccoons by radio. Journal of Wildlife Management, 28, 363–368.

Elowe, K.D. & Dodge, W.E. (1989) Factors affecting black bear reproductive success and cub survival. Journal of Wildlife Management, 53, 962–968.

Erlinge, S. (1977) Spacing strategy in stoat, Mustela erminea. Oikos, 28, 32–42.

Escherich, P.C. (1981) Social biology of the bushy-tailed woodrat, Neotoma cinerea. University of California Press.

Estes-Zumpf, W.A. & Rachlow, J.L. (2009) Natal dispersal by pygmy rabbits (Brachylagus idahoensis). Journal of Mammalogy, 90, 363–372.

Evans, F.C.& Holdenried, R. (1943) A population study of the Beechey ground squirrel in central California. Journal of Mammalogy, 24, 231–238.

Ferguson, J., Nel, J. & Wet, M.J. (1983) Social organization and movement patterns of black-backed jackals (Canis mesomelas) in South Africa. Journal of Zoology, 199, 487–502.

Fernandez-Duque, E. (2009) Natal dispersal in monogamous owl monkeys (Aotus azarai) of the Argentinean Chaco. Behaviour, 146, 583–600.

Ferreras, P., Delibes, M., Palomares, F., Fedriani, J.M., Calzada, J. & Revilla, E. (2004) Proximate and ultimate causes of dispersal in the Iberian lynx (Lynx pardinus). Behavioral Ecology, 15, 31–40.

Fisher, D.O. (2005) Population density and presence of the mother are related to natal dispersal in male and female Antechinus stuartii. Australian Journal of Zoology, 53, 103–111.

Fisher, D.O., Lambin, X. & Yletyinen, S.M. (2009) Experimental translocation of juvenile water voles in a Scottish lowland metapopulation. Population Ecology, 51, 289–295.

Fisler, G.F. (1962) Homing in the California vole, Microtus californicus. American Midland Naturalist, 68, 357–368.

Fisler, G.F. (1966) Homing in the western harvest mouse, Reithrodontomys megalotis. Journal of Mammalogy, 47, 53–58.

Fitch, H.S. (1958) Home ranges, territories, and seasonal movements of vertebrates of the Natural History Reservation. University of Kansas Museum of Natural History, 11, 1–54.

Flagstad, O., Brøseth, H., Johansson, M., Wärdig, C. & Ellegren, H. (2008) DNA-based monitoring of the Scandinavian wolverine population 2000-2007. In NINA Rapport.

Frame, G. (1984) Cheetah. In The Encyclopedia of Mammals (1st ed., pp. 40–43).

French, N.R., McBride, R. & Detmer, J. (1965) Fertility and population density of the black-tailed jackrabbit. Journal of Wildlife Management, 29, 14–26.

Fritts, S.H. (1983) Record dispersal by a wolf from Minnesota. Journal of Mammalogy, 64, 166–167.

Fritts, S.H. & Mech, L.D. (1981) Dynamics, movements, and feeding ecology of a newly protected wolf population in northwestern Minnesota. Wildlife Monographs, 80, 3–79.

Fritts, S. H. (1984) Record dispersal by a wolf from Minnesota. J. Mammal., 64: 166-167.

Fritzell, E.K. (1978) Aspects of raccoon (Procyon lotor) social organization. Canadian Journal of Zoology, 56, 260–271.

Fuller, T., Mills, M., Borner, M., Laurenson, M. & Kat, P. (1992) Long-distance dispersal by African wild dogs in East and South Africa (Lycaon pictus). Journal of African Zoology, 106, 1–20.

Funston, P.J., Mills, M.G.L. & Van Heezik, Y. (2003) Reduced dispersal and opportunistic territory acquisition in male lions (Panthera leo). Journal of Zoology, 259, 131–142.

Gaillard, J.M., Hewison, A.J.M., Kjellander, P., Pettorelli, N., Bonenfant, C., Van Moorter, B., Liberg, O., Andren, H., Van Laere, G., & Klein, F. (2008) Population density and sex do not influence fine-scale natal dispersal in roe deer. Proceedings of the Royal Society B: Biological Sciences, 275, 2025–2032.

Gardner, C.L., Ballard, W.B. & E. C. Hellgren (1986) Long-distance movement by an adult wolverine. Journal of Mammalogy, 67, 603–605.

Garrett, M.G. & Franklin, W.L. (1988) Behavioral ecology of dispersal in the black-tailed prairie dog. Journal of Mammalogy, 69, 236–250.

Gehrt, S.D. & Fritzell, E.K. (1998) Duration of familial bonds and dispersal patterns for raccoons in south Texas. Journal of Mammalogy, 79, 859–872.

Gentry, J.B. (1964) Homing in the old-field mouse. Journal of Mammalogy, 45, 276–283.

Gerell, R. (1970) Home ranges and movements of the mink (Mustela vison) in southern Sweden. Oikos, 21, 160–173.

Gese, E.M. & Mech, L.D. (1991) Dispersal of wolves (Canis lupus) in northeastern Minnesota, 1969-1989. Canadian Journal of Zoology, 69, 2946–2955.

Gillette, L.N. (1980) Movement patterns of radio-tagged opossums in Wisconsin. American Midland Naturalist, 104, 1–12.

Gillis, E.A. & Krebs, C.J. (1999) Natal dispersal of snowshoe hares during a cyclic population increase. Journal of Mammalogy, 80, 933–939.

Glass, G.E., Korch, G.W., Gomez, J.E. & Childs, J.E. (1991) Using exotic antigens to measure reproduction and dispersal in Peromyscus leucopus. Canadian Journal of Zoology, 69, 528–530.

Glenn, L.P. & Miller, L.H. (1980) Seasonal movements of an Alaska Peninsula brown bear population. In Bears: Their Biology and Management (pp. 307–312).

Green, J.S. & Flinders, J.T. (1980) Brachylagus idahoensis. Mammalian Species, (125), 1–4.

Griffo Jr, J.V. (1961) A study of homing in the cotton mouse, Peromyscus gossypinus. American Midland Naturalist, 65, 257–289.

Gursky, S. (2010) Dispersal patterns in Tarsius spectrum. International Journal of Primatology, 31, 117–131.

Hackett, D.F. (1987) Dispersal of yearling Columbian ground squirrels. PhD thesis, University of Alberta.

Halley, D., Vandewalle, M., Mari, M. & Taolo, C. (2002) Herd-switching and long-distance dispersal in female African buffalo (Syncerus caffer). African Journal of Ecology, 40, 97–99.

Hamilton, W., Cook, A.H. & Hamilton, W. (1955) The biology and management of the fisher in New York. New York Fish and Game Journal, 2, 13–35.

Harris, J.H., Leitner, P. & Edwards, C.W. (2005) Long-distance movements of juvenile Mohave ground squirrels, Spermophilus mohavensis. Southwestern Naturalist, 50, 188–196.

Harris, S. & Trewhella, W.J. (1988) An analysis of some of the factors affecting dispersal in an urban fox (Vulpes vulpes) population. Journal of Applied Ecology, 25, 409–422.

Harrison, D.J. (1992) Dispersal characteristics of juvenile coyotes in Maine. Journal of Wildlife Management, 56, 128–138.

Hedlund, J.D. (1975) Tagging mule deer fawns in South-Central Washington, 1969–1974. Northwest Science, 49, 153–157.

Hemker, T.P., Lindzay, F.G., & Ackerman, BB. (1984) Population characteristics and movement patterns of cougars in Southern Utah. Journal of Wildlife Management, 48, 1275–1284.

Henshaw, R.E. & Stephenson, R.O. (1974) Homing in the Gray Wolf (Canis lupus). Journal of Mammalogy, 55, 234–237.

Herrera, E.A. (1992) Growth and dispersal of capybaras (Hydrochaeris hydrochaeris) in the Llanos of Venezuela. Journal of Zoology, 228, 307–316.

Hickie, P. & Whitlock, S.C. (1940) Cottontails in Michigan. Game Division, Michigan Department of Conservation.

Hoffmeister, D.F. (1977) Long-distance homing of a cottontail. American Midland Naturalist, 97, 221–224.

Holekamp, K.E. (1984) Natal dispersal in Belding's ground squirrels (Spermophilus beldingi). Behavioral Ecology and Sociobiology, 16, 21–30.

Hoogland, J.L. (1999) Philopatry, dispersal, and social organization of Gunnison's prairie dogs. In Kangaroos, Wallabies and Rat-kangaroos (pp. 243–251). G. Grigg, P. Jarman, & I. Hume (Eds). Surrey Beatty & Sons Pty Limited, NSW, Australia.

Jacques, C.N. & Jenks, J.A. (2007) Dispersal of yearling pronghorns in western South Dakota. Journal of Wildlife Management, 71, 177–182.

Jacquot, J.J. & Vessey, S.H. (1995) Influence of the natal environment on dispersal of white-footed mice. Behavioral Ecology and Sociobiology, 37, 407–412.

Janmaat, K.R.L., Olupot, W., Chancellor, R.L., Arlet, M.E. & Waser, P.M. (2009) Long-term site fidelity and individual home range shifts in Lophocebus albigena. International Journal of Primatology, 30, 443–466.

Jenkins, D.G., Brescacin, C.R., Duxbury, C.V., Elliott, J.A., Evans, J.A., Grablow, K.R., et al. (2007) Does size matter for dispersal distance? Global Ecology and Biogeography, 16, 415–425.

Jensen, B. (1973) Movements of the red fox (Vulpes vulpes) in Denmark investigated by marking and recovery. Danish Review of Game Biology, 8, 1–20.

Johnson, C.N. & Payne, A. (2002) Sex-biased dispersal in the rufous bettong, Aepyprymnus rufescens. Australian Mammalogy, 24, 233–239.

Johnson, C.A., Fryxell, J.M., Thompson, I.D. & Baker, J.A. (2009) Mortality risk increases with natal dispersal distance in American martens. Proceedings of the Royal Society B: Biological Sciences, 276, 3361–3368.

Johnson, S.A., Walker, H.D. & Hudson, C.M. (2010) Dispersal characteristics of juvenile bobcats in south-central Indiana. Journal of Wildlife Management, 74, 379–385.

Jones, J.L. (1991) Habitat use of fisher in north central Idaho. MS thesis, University of Idaho, Moscow.

Jones, W.T. (1987) Dispersal patterns in kangaroo rats (Dipodomys spectabilis). In Mammalian Dispersal Patterns: The Effects of Social Structure on Population Genetics (pp. 119–127). B.D. Chepko-Sade & Z.T. Halpin (Eds). University of Chicago Press, Chicago, Illinois, USA.

Jones, W.T. (1989) Dispersal distance and the range of nightly movements in Merriam's kangaroo rats. Journal of Mammalogy, 70, 27–34.

Jones, W.T., Waser, P.M., et al. (1988) Philopatry, dispersal, and habitat saturation in the banner-tailed kangaroo rat, Dipodomys spectabilis. Ecology, 69, 1466–1473.

Juškaitis, R. (1997) Ranging and movement of the common dormouse (Muscardinus avellanarius) in Lithuania. Acta Theriologica, 42, 113–122.

Juškaitis, R. (2008) The Common Dormouse (Muscardinus avellanarius). Institute of Ecology of Vilnius University Publishers, Vilnius.

Kamler, J.F., Gipson, P.S., et al. (2000) Dispersal characteristics of young bobcats from northeastern Kansas. Wildlife Society Bulletin, 28, 543–546.

Katzner, T.E. & Parker, K.L. (1998) Long-distance movements from established burrow sites by pygmy rabbits (Brachylagus idahoensis) in southwestern Wyoming. Northwestern Naturalist, 79, 72–74.

Keane, B. (1990) Dispersal and inbreeding avoidance in the white-footed mouse (Peromyscus leucopus). Animal Behaviour, 40, 143–152.

Keith, L.B. & Waring, J. (1956) Evidence of orientation and homing in snowshoe hares. Canadian Journal of Zoology, 34, 579–581.

Kernohan, B.J., Jenks, J.A. & Naugle, D.E. (1994) Refuge, South Dakota. Prairie Naturalist, 26, 4.

Knick, S.T. (1990) Ecology of bobcats relative to exploitation and a prey decline in southeastern Idaho. Wildlife Monographs, 108, 3–42.

Knick, S.T. & Bailey, T.N. (1986) Long-distance movements by two bobcats from southeastern Idaho. American Midland Naturalist, 115, 222–223.

Kojola, I., Aspi, J., et al. (2006) Dispersal in an expanding wolf population in Finland. Journal of Mammalogy, 87, 281–286.

Koontz, F., Horwich, R., Saqui, E., Saqui, H., Glander, K., Koontz, C. & Westrom, W. (1994) Reintroduction of black howler monkeys (Alouatta pigra) into the Cockscomb Basin Wildlife Sanctuary, Belize. In AZA Annual Conference Proceedings (pp. 104–111).

Koopman, M.E., Cypher, B.L., et al. (2000) Dispersal patterns of San Joaquin kit foxes (Vulpes macrotis mutica). Journal of Mammalogy, 81, 213–222.

Laack, L.L. (1991) Ecology of the ocelot (Felis pardalis) in south Texas. MS thesis, Texas A & I University.

Lackey, J.A., Huckaby, D.G., et al. (1985) Peromyscus leucopus. Mammalian Species, 247, 1–10.

Lambin, X. (1994) Natal philopatry, competition for resources, and inbreeding avoidance in Townsend's voles (Microtus townsendii). Ecology, 75, 224–235.

Lariviere, S. & Seddon, P.J. (2001) Vulpes rueppelli. Mammalian Species, 678, 1–5.

Lariviere, S. & Walton, L.R. (1997) Lynx rufus. Mammalian Species, 563, 1–8.

Layne, J.N. & Glover, D. (1977) Home range of the armadillo in Florida. Journal of Mammalogy, 58, 411–413.

Lechleitner, R. (1958) Movements, density, and mortality in a black-tailed jack rabbit population. Journal of Wildlife Management, 22, 371–384.

Letnic, M. (2002) Long distance movements and the use of fire mosaics by small mammals in the Simpson Desert, central Australia. Australian Mammalogy, 23, 125–134.

Lidicker Jr, W.Z. & Patton, J.L. (1987) Patterns of dispersal and genetic structure in populations of small rodents. In Mammalian Dispersal Patterns: The Effects of Social Structure on Population Genetics (pp. 144). B.D. Chepko-Sade & Z.T. Halpin (Eds). University of Chicago Press, Chicago.

Lindzay, F.G., Van Sickle, W.D., et al. (1994) Cougar population dynamics in southern Utah. Journal of Wildlife Management, 58, 619–624.

Lindzey, P.G. (1978) Movement patterns of badgers in northwestern Utah. Journal of Wildlife Management, 42, 418–422.

Linklater, W.L. & Cameron, E.Z. (2009) Social dispersal but with philopatry reveals incest avoidance in a polygynous ungulate. Animal Behaviour, 77, 1085–1093.

Lofroth, E.C. (1993) Scale dependent analyses of habitat selection by marten in the sub-boreal spruce biogeoclimatic zone, British Columbia. Thesis, Simon Fraser University, Burnaby, British Columbia, Canada.

Logan, K.A., Irwin, L.L., et al. (1986) Characteristics of a hunted mountain lion population in Wyoming. Journal of Wildlife Management, 50, 648–654.

Long, E.S., Diefenbach, D.R., et al. (2005) Forest cover influences dispersal distance of white-tailed deer. Journal of Mammalogy, 86, 623–629.

Lynch, G.M. (1967) Long-range movement of a raccoon in Manitoba. Journal of Mammalogy, 48, 659–660.

Macdonald, D.W. & Courtenay, O. (1996) Enduring social relationships in a population of crab-eating zorros, Cerdocyon thous, in Amazonian Brazil. Journal of Zoology, 239, 329–355.

Macpherson, A. (1968) Apparent recovery of translocated arctic fox. Canadian Field-Naturalist, 82, 287–289.

Maehr, D.S., Land, E.D., Shindle, D.B., Bass, O.L. & Hoctor, T.S. (2002) Florida panther dispersal and conservation. Biological Conservation, 106, 187–197.

Maehr, D.S., Land, E. & Roof, J. (1999) Social ecology of Florida panthers. NCASI Technical Bulletin, National Geographic Research, 7, 321.

Magoun, A.J. (1985) Population characteristics, ecology, and management of wolverines in northwestern Alaska. PhD thesis, University of Alaska, Fairbanks.

Maier, T.J. (2002) Long-distance movements by female white-footed mice (Peromyscus leucopus) in extensive mixed-wood forest. Canadian Field-Naturalist, 116, 108–111.

Marmet, J., Pisanu, B. & Chapuis, J.L. (2011) Natal dispersal of introduced Siberian chipmunks (Tamias sibiricus) in a suburban forest. Journal of Ethology, 29, 23–29.

Martin, J.M. & Heske, E.J. (2005) Juvenile dispersal of Franklin's ground squirrel (Spermophilus franklinii) from a prairie “island”. American Midland Naturalist, 153, 444–449.

Matthews, S., Higley, J.M., Green, R.E., Rennie, K.M. & Goddard, C.A. (2009) Juvenile fisher dispersal patterns on Hoopa Valley Reservation, California. Preliminary report to US Fish and Wildlife Service Yreka Field Office in partial fulfillment of Agreement Number 813338G015.

McCarley, H. (1966) Annual cycle, population dynamics and adaptive behavior of Citellus tridecemlineatus. Journal of Mammalogy, 47, 294–316.

McCoy, J.E., Hewitt, D.G., et al. (2005) Dispersal by yearling male white-tailed deer and implications for management. Journal of Wildlife Management, 69, 366–376.

McGuire, B., Getz, L.L., et al. (1993) Natal dispersal and philopatry in prairie voles (Microtus ochrogaster) in relation to population density, season, and natal social environment. Behavioral Ecology and Sociobiology, 32, 293–302.

McLellan, B.N. & Hovey, F.W. (2001) Natal dispersal of grizzly bears. Canadian Journal of Zoology, 79, 838–844.

McNutt, J.W. (1996) Sex-biased dispersal in African wild dogs, Lycaon pictus. Animal Behaviour, 52, 1067–1077.

Mech, L.D. (1977) Record movement of a Canadian lynx. Journal of Mammalogy, 58, 676–677.

Mech, L.D. (1987) Age, season, distance, direction, and social aspects of wolf dispersal from a Minnesota pack. In Mammalian Dispersal Patterns: The Effects of Social Structure on Population Genetics (pp. 55–74). B.D. Chepko-Sade & Z.T. Halpin (Eds). University of Chicago Press, Chicago, Illinois, USA.

Mech, L.D., Fritts, S.H. & Wagner, D. (1995) Minnesota wolf dispersal to Wisconsin and Michigan. American Midland Naturalist, 133, 368–370.

Melquist, W.E. & Hornocker, H.G. (1983) Ecology of river otters in west central Idaho. Wildlife Monographs, 83, 1–60.

Messick, J.P. & Hornocker, M.G. (1981) Ecology of the badger in southwestern Idaho. Wildlife Monographs, 76, 3–53.

Michener, G.R. & Michener, D.R. (1977) Population structure and dispersal in Richardson's ground squirrels. Ecology, 58, 359–368.

Michener, G.R. & Koeppl, J.W. (1985) Spermophilus richardsonii. Mammalian Species, 243, 1–8.

Mihok, S., Lawton, T. & Schwartz, B. (1988) Fates and movements of meadow voles (Microtus pennsylvanicus) following a population decline. Canadian Journal of Zoology, 66, 323–328.

Miller, B., Reading, R.P. & Forrest, S. (1996) Prairie Night: Black-Footed Ferrets and the Recovery of Endangered Species. Smithsonian Institution Press.

Miller, S.D. & Ballard, W.B. (1982) Homing of transplanted Alaskan brown bears. Journal of Wildlife Management, 46, 869–876.

Milner-Gulland, E.J., et al. (2006) Application of IUCN red listing criteria at the regional and national levels: a case study from Central Asia. Biodiversity and Conservation, 15, 1873–1886.

Mitchell, J.L. (1961) Mink movements and populations on a Montana river. Journal of Wildlife Management, 25, 48–54.

Mockrin, M.H. (2010) Duiker demography and dispersal under hunting in Northern Congo. African Journal of Ecology, 48, 239–247.

Murie, O.J. & Murie, A. (1931) Travels of Peromyscus. Journal of Mammalogy, 12, 200–209.

Murray, J.L. & Gardner, G.L. (1997) Leopardus pardalis. Mammalian Species, 548, 1–10.

Murray, M.G. (1982) Home range, dispersal and the clan system of impala. African Journal of Ecology, 20, 253–269.

Nelson, M.E. & Mech, L.D. (1992) Dispersal in female white-tailed deer. Journal of Mammalogy, 73, 891–894.

Nelson, M.E. & Mech, L.D. (1984) Home-range formation and dispersal of deer in northeastern Minnesota. Journal of Mammalogy, 65, 567–575.

Nelson, M.E. & Mech, L.D. (1987) Demes within a northeastern Minnesota deer population. In Mammalian Dispersal Patterns: The Effects of Social Structure on Population Genetics (pp. 27–40). B.D. Chepko-Sade & Z.T. Halpin (Eds). University of Chicago Press, Chicago.

Nicholson, K.L., Ballard, W.B., McGee, B.K. & Whitlaw, H.A. (2007) Dispersal and extraterritorial movements of swift foxes (Vulpes velox) in northwestern Texas. Western North American Naturalist, 67, 102–108.

Nicholson, W.S., Hill, E.P., et al. (1985) Denning, pup-rearing, and dispersal in the gray fox in east-central Alabama. Journal of Wildlife Management, 49, 33–37.

Nishida, T. (1966) A sociological study of solitary male monkeys. Primates, 7, 141–204.

Nixon, C.M. (1994) Behavior, dispersal, and survival of male white-tailed deer in Illinois. Biological Notes, 139, 1–30.

Nowell, K. & Jackson, P. (1996) Wild Cats - Status Survey and Conservation Action Plan. In: IUCN, (Ed). IUCN, Gland, Switzerland.

O'Donoghue, M., Boutin, S., Krebs, C.J. & Hofer, E.J. (1997) Numerical responses of coyotes and lynx to the snowshoe hare cycle. Oikos, 80, 150–162.

O'Farrell, T. (1984) Conservation of the endangered San Joaquin kit fox, Vulpes macrotis mutica, on the Naval Petroleum Reserves, California. Acta Zoologica Fennica, 172, 207–208.

O'Farrell, T.P. (1965) Home range and ecology of snowshoe hares in interior Alaska. Journal of Mammalogy, 46, 406–418.

Oksanen, T., Schneider, M., Rammul, Ü., Hambäck, P. & Aunapuu, M. (1999) Population fluctuations of voles in North Fennoscandian tundra: contrasting dynamics in adjacent areas with different habitat composition. Oikos, 86, 463–478.

Olson, G.S. & Van Horne, B. (1998) Dispersal patterns of juvenile Townsend's ground squirrels in southwestern Idaho. Canadian Journal of Zoology, 76, 2084–2089.

Ortega, J.C. (1988) The behavioral ecology and natural history of the rock squirrel (Spermophilus variegatus) in southeastern Arizona. PhD thesis, University of Colorado.

Ostfeld, R.S. & Manson, R.H. (1996) Long-distance homing in meadow voles, Microtus pennsylvanicus. Journal of Mammalogy, 87, 870–873.

Parsons, B.C., Short, J.C. & Calver, M.C. (2002) Evidence for male-biased dispersal in a reintroduced population of burrowing bettongs (Bettongia lesueur) at Heirisson Prong, Western Australia. Australian Mammalogy, 24, 219–224.

Pasitschniak-Arts, M. & Lariviere, S. (1995) Gulo gulo. Mammalian Species, 499, 1–10.

Payne, N.F. (1975) Unusual movements of Newfoundland black bears. Journal of Wildlife Management, 39, 812–813.

Peacock, M.M. & Smith, A.T. (1997) The effect of habitat fragmentation on dispersal patterns, mating behaviour and genetic variation in a pika (Ochotona princeps) metapopulation. Oecologia, 112, 524–533.

Pearson, A. (1972) Population characteristics of the northern interior grizzly in the Yukon Territory, Canada. In Bears: Their Biology and Management (pp. 32–35).

Peterson, R.O., Woolington, J.D. & Bailey, T.N. (1984) Wolves of the Kenai Peninsula, Alaska. Wildlife Monographs, 88, 3–52.

Phillips, R.L., Storm, G.L. et al. (1972) Dispersal and mortality of red foxes. Journal of Wildlife Management, 36, 237–244.

Phillips, R. & Mech, L.D. (1970) Homing behavior of a red fox. Journal of Mammalogy, 51, 621–622.

Pinter-Wollman, N.M. (2008) The effects of translocation on the behavior of African elephants (Loxodonta africana). PhD thesis, University of California.

Pocock, M.J.O., Hauffe, H.C. & Searle, J.B. (2005) Dispersal in house mice. Biological Journal of the Linnean Society, 84, 565–583.

Poole, K.G. (1997) Dispersal patterns of lynx in the Northwest Territories. Journal of Wildlife Management, 61, 497–505.

Price, M.V., Kelly, P.A. et al. (1994) Distances moved by Stephens' kangaroo-rat (Dipodomys stephensi merriam) and implications for conservation. Journal of Mammalogy, 75, 929–939.

Priddel, D., Wellard, G. & Shepherd, N. (1988) Movements of sympatric red kangaroos (Macropus rufus) and western grey kangaroos (Macropus fuliginosus) in western New South Wales. Wildlife Research, 15, 339–346.

Priewert, F.W. (1961) Record of an extensive movement by a raccoon. Journal of Mammalogy, 42, 113–113.

Priotto, J., Steinmann, A. et al. (2004) Juvenile dispersal in Calomys venustus. Acta Oecologica, 25, 205–210.

Pullianen, E. (1974) Seasonal movements of moose in Europe. Naturaliste Canadien, 101, 379–392.

Quaglietta, L. (2011) Ecology and behaviour of the Eurasian otter (Lutra lutra) in a Mediterranean area (Alentejo, Portugal). PhD thesis, La Sapienza, Rome.

Quanstrom, W.R. (1971) Behaviour of Richardson's ground squirrel (Spermophilus richardsonii richardsonii). Animal Behaviour, 19, 646–652.

Quirici, V., Faugeron, S., Hayes, L.D. & Ebensperger, L.A. (2011) The influence of group size on natal dispersal in the communally rearing and semifossorial rodent, Octodon degus. Behavioral Ecology and Sociobiology, 65, 1–12.

Rado, R., Wollberg, Z. & Terkel, J. (1992) Dispersal of young mole rats (Spalax ehrenbergi) from the natal burrow. Journal of Mammalogy, 73, 885–890.

Rehmeier, R.L., Kaufman, G.A. & Kaufman, D.W. (2004) Long-distance movements of the deer mouse in tallgrass prairie. Journal of Mammalogy, 85, 562–568.

Ribble, D.O. (1992) Dispersal in a monogamous rodent, Peromyscus californicus. Ecology, 73, 859–866.

Robinette, W.L. (1966) Mule deer home range and dispersal in Utah. Journal of Wildlife Management, 30, 335–349.

Robinson, W.B. & Grant, E.F. (1958) Comparative movements of bobcats and coyotes as disclosed by tagging. Journal of Wildlife Management, 22, 117–122.

Rogers, L.L. (1987a) Effects of food supply and kinship on social behavior, movements, and population growth of black bears in northeastern Minnesota. Wildlife Monographs, 97, 3–72.

Rogers, L.L. (1987b) Factors influencing dispersal. In Mammalian Dispersal Patterns: The Effects of Social Structure on Population Genetics (p. 75). B.D. Chepko-Sade & Z.T. Halpin (Eds). University of Chicago Press, Chicago.

Rogers, L.L. (1988) Homing tendencies of large mammals: a review. In Translocation of Wild Animals (pp. 123–137). N.A. Brown & R.D. Brown (Eds). Wisconsin Humane Society and Caesar Kleberg Wildlife Research Institute, Madison, Wisconsin.

Rogers, M.J. (1974) Movements and reproductive success of black bears introduced into Arkansas. In Proceedings of the Annual Conference of the Southeastern Association of Fish and Wildlife Agencies (pp. 307–308).

Rongstad, O.J. (1965) A life history study of thirteen-lined ground squirrels in southern Wisconsin. Journal of Mammalogy, 46, 76–87.

Rood, J.P. (1987) Dispersal and intergroup transfer in the dwarf mongoose. In Mammalian Dispersal Patterns: The Effects of Social Structure on Population Genetics (pp. 85–103). B.D. Chepko-Sade & Z.T. Halpin (Eds). University of Chicago Press, Chicago.

Ross, P.I. & Jalotsky, M.G. (1992) Characteristics of a hunted population of cougars in southwestern Alberta. Journal of Wildlife Management, 56, 417–426.

Roy, K.D. (1991) Ecology of reintroduced fishers in the Cabinet Mountains of northwest Montana. MS thesis, University of Montana.

Roze, U. (1989) The North American Porcupine, 2nd edn. Smithsonian Institution, Washington, D.C.

Ruth, T.K., Logan, K.A., Sweanor, L.L., Hornocker, M.G. & Temple, L.J. (1998) Evaluating cougar translocation in New Mexico. Journal of Wildlife Management, 62, 1264–1275.

Rutherglen, R. & Herbison, B. (1977) Movements of nuisance black bears (Ursus americanus) in southeastern British Columbia. Canadian Field-Naturalist, 91, 419–422.

Sandell, M., Agrell, J. et al. (1990) Natal dispersal in relation to population density and sex ratio in the field vole, Microtus agrestis. Oecologia, 83, 145–149.

Saunders, J.K.J. (1963) Movements and activities of the lynx in Newfoundland. Journal of Wildlife Management, 27, 390–400.

Schwartz, C.W. (1941) Home range of the cottontail in central Missouri. Journal of Mammalogy, 22, 386–392.

Seidel, D. (1961) Homing in the eastern chipmunk. Journal of Mammalogy, 42, 256–257.

Sheldon, W.G. (1953) Returns on banded red and gray foxes in New York State. Journal of Mammalogy, 34, 125–126.

Shields, P.W. (1960) Movement patterns of brush rabbits in northwestern California. Journal of Wildlife Management, 24, 381–386.

Shrader, A.M. & Owen-Smith, N. (2002) The role of companionship in the dispersal of white rhinoceroses (Ceratotherium simum). Behavioral Ecology and Sociobiology, 52, 255–261.

Sillero-Zubiri, C. & Macdonald, D.W. (1997) The Ethiopian wolf - Status survey and conservation action plan. In: The African Canid: Ecology, Behavior, and Conservation (ISCS Group, Ed.). IUCN, Gland, Switzerland.

Sillero-Zubiri, C., Hoffmann, M. & Macdonald, D.W. (2004) Canids: Foxes, wolves, jackals and dogs. Status survey and conservation action plan. In: The Canid Specialist Group Report (ISCS Group, Ed.). IUCN, Gland, Switzerland and Cambridge, UK.

Slough, B.G. (1989) Movements and habitat use by transplanted marten in the Yukon Territory. Journal of Wildlife Management, 53, 991–997.

Slough, B.G. & Mowat, G. (1996) Lynx population dynamics in an untrapped refugium. Journal of Wildlife Management, 60, 946–961.

Smith, A.T. (1987) Population structure of pikas: Dispersal versus philopatry. In: Mammalian Dispersal Patterns: The Effects of Social Structure on Population Genetics (Chepko-Sade, B.D. & Halpin, Z.T., Eds), pp. 128–142. University of Chicago Press, Chicago.

Smith, A.A. (1997) Dispersal and movements in a Swedish willow grouse (Lagopus lagopus) population. Wildlife Biology, 3, 279–285.

Smith, A.T. & Ivins, B.L. (1983) Colonization in a pika population: Dispersal vs philopatry. Behavioral Ecology and Sociobiology, 13, 37–47.

Smith, J.L.D. (1993) The role of dispersal in structuring the Chitwan tiger population. Behaviour, 124, 165–195.

Smith, M.H. (1968) Dispersal of the old-field mouse, Peromyscus polionotus. Bulletin of the Georgia Academy of Science, 26, 45–51.

Soderquist, T. & Lill, A. (1995) Natal dispersal and philopatry in the carnivorous marsupial, Phasogale tapoatafa (Dasyuridae). Ethology, 99, 297–312.

Steen, H. (1994) Low survival of long-distance dispersers of the root vole (Microtus oeconomus). Annales Zoologici Fennici, 31, 271–274.

Stickel, L.F. (1949) An experiment on Peromyscus homing. American Midland Naturalist, 41, 659–664.

Stoddart, D.M. (1970) Individual range, dispersion and dispersal in a population of water voles (Arvicola terrestris (L.)). Journal of Animal Ecology, 39, 403–425.

Støen, O.G., Zedrosser, A., Sæbø, S. & Swenson, J.E. (2006) Inversely density-dependent natal dispersal in brown bears (Ursus arctos). Oecologia, 148, 356–364.

Storm, G.L., Andrews, R.D. et al. (1976) Morphology, reproduction, dispersal and mortality of midwestern red fox populations. Wildlife Monographs, 49, 1–72.

Stratman, M.R., Alden, C.D., Pelton, M.R. & Sunquist, M.E. (2001) Long distance movement of a Florida black bear in the southeastern coastal plain. Ursus, 12, 55–58.

Stuewer, F.W. (1943) Raccoons: Their habits and management in Michigan. Ecological Monographs, 13, 203–257.

Sullivan, E.G. (1956) Gray fox reproduction, denning, range, and weights in Alabama. Journal of Mammalogy, 37, 346–351.

Sunquist, M.E. (1983) Dispersal of three radiotagged leopards. Journal of Mammalogy, 64, 337–341.

Sutor, A. (2008) Dispersal of the alien raccoon dog (Nyctereutes procyonoides) in southern Brandenburg, Germany. European Journal of Wildlife Research, 54, 321–326.

Svendsen, G.E. (1974) Behavioral and environmental factors in the spatial distribution and population dynamics of a yellow-bellied marmot population. Ecology, 55, 760–771.

Sweanor, L.L., Logan, K.A. et al. (2000) Cougar dispersal patterns, metapopulation dynamics, and conservation. Conservation Biology, 14, 798–808.

Swihart, R.K. (1992) Home-range attributes and spatial structure of woodchuck populations. Journal of Mammalogy, 73, 604–618.

Tabatabai, F. & Kennedy, M. (1989) Movements of relocated raccoons (Procyon lotor) in western Tennessee. Journal of the Tennessee Academy of Science, 64, 221–224.

Taylor, J.M. & Calaby, J.H. (1988) Rattus fuscipes. Mammalian Species, 298, 1–8.

Teferi, T. & Millar, J. (1993) Long distance homing by the deer mouse, Peromyscus maniculatus. Canadian Field-Naturalist, 107, 109–111.

Tegelström, H. & Hansson, L. (1987) Evidence of long distance dispersal in the common shrew (Sorex araneus). Zeitschrift für Säugetierkunde, 52, 52–54.

Telfer, S., Piertney, S., Dallas, J., Stewart, W., Marshall, F., Gow, J. & Lambin, X. (2003) Parentage assignment detects frequent and large-scale dispersal in water voles. Molecular Ecology, 12, 1939–1949.

Thompson, D. & Jenks, J. (2010) Dispersal movements of subadult cougars from the Black Hills: The notions of range expansion and recolonization. Ecosphere, 1, 1–18.

Tierson, W.C., Mattfeld, G.F., Sage Jr, R.W. & Behrend, D.F. (1985) Seasonal movements and home ranges of white-tailed deer in the Adirondacks. Journal of Wildlife Management, 49, 760–769.

Tileston, J.V. & Lechleitner, R.R. (1966) Some comparisons of the black-tailed and white-tailed prairie dogs in north-central Colorado. American Midland Naturalist, 75, 292–316.

Trewhella, W.J., Harris, S. et al. (1988) Dispersal distance, home-range size and population density in the red fox (Vulpes vulpes): A quantitative analysis. Journal of Applied Ecology, 25, 423–434.

Trombulak, S.C. (1987) Life history of the cascade golden-mantled ground squirrel (Spermophilus saturatus). Journal of Mammalogy, 68, 544–554.

Truvé, J., Lemel, J. & Söderberg, B. (2004) Dispersal in relation to population density in wild boar (Sus scrofa). Galemys, 16, 75–82.

Tullar, B.F. & Berchielli, L.T. (1982) Comparison of red foxes and gray foxes in central New York with respect to certain features of behavior, movement and mortality. New York Fish and Game Journal, 29, 127–133.

Urban, D. (1970) Raccoon populations, movement patterns, and predation on a managed waterfowl marsh. Journal of Wildlife Management, 34, 372–382.

Van Camp, J. & Gluckie, R. (1979) A record long-distance move by a wolf (Canis lupus). Journal of Mammalogy, 60, 236–237.

Van Vuren, D. (1990) Dispersal of yellow-bellied marmots. PhD thesis, University of Kansas.

VanDruff, L.W. (1969) Movements of opossums in marsh and wetland habitats. In: Transactions of Northeast Fish and Wildlife Conference (White Sulphur Springs, West Virginia, USA, 1969), pp. 81–89. Northeast Section of the Wildlife Society, Amherst, Massachusetts, USA.

Vangen, K.M., Persson, J. et al. (2001) Characteristics of dispersal in wolverines. Canadian Journal of Zoology, 79, 1641–1649.

Vaughan, T.A. (1963) Movements made by two species of pocket gophers. American Midland Naturalist, 69, 367–372.

Wabakken, P., Sand, H., Kojola, I., Zimmermann, B., Arnemo, J.M., Pedersen, H.C. & Liberg, O. (2007) Multistage, long-range natal dispersal by a global positioning system-collared Scandinavian wolf. Journal of Wildlife Management, 71, 1631–1634.

Wahlström, L. & Liberg, O. (1995) Patterns of dispersal and seasonal migration in roe deer (Capreolus capreolus). Journal of Zoology, 235, 455–467.

Ward, R.M.P. & Krebs, C.J. (1985) Behavioural responses of lynx to declining snowshoe hare abundance. Canadian Journal of Zoology, 63, 2817–2824.

Wauters, L. & Dhondt, A.A. (1993) Immigration pattern and success in red squirrels. Behavioral Ecology and Sociobiology, 33, 159–167.

Wauters, L.A., Verbeylen, G., Preatoni, D., Martinoli, A. & Matthysen, E. (2010) Dispersal and habitat cuing of Eurasian red squirrels in fragmented habitats. Population Ecology, 52, 527–536.

Weir, R.D., Corbould, F.B. & Apps, C.D. (2006) Density of fishers in the Sub-boreal Spruce biogeoclimatic zone of British Columbia. Northwestern Naturalist, 87, 118–127.

Wiggett, D.R. & Boag, D.A. (1989) Intercolony natal dispersal in the Columbian ground squirrel. Canadian Journal of Zoology, 67, 42–50.

Wolff, J.O. & Lidicker, W.Z. Jr. (1980) Population ecology of the taiga vole (Microtus xanthognathus) in interior Alaska. Canadian Journal of Zoology, 58, 1800–1812.

Woodruff, R. A., and B. L. Keller. (1982) Dispersal, daily activity, and home range of coyotes in southeastern Idaho. Northwest Science 56(3): 199-207.

Wrigley, R.E. & Hatch, D.R.M. (1976) Arctic fox migrations in Manitoba. Arctic, 29, 147–158.

York, E.C. (1996) Fisher population dynamics in north-central Massachusetts. PhD thesis, University of Massachusetts at Amherst.

Yott, A., Rosatte, R., Schaefer, J.A., Hamr, J. & Fryxell, J.M. (2011) Movement and spread of a founding population of reintroduced elk (Cervus elaphus) in Ontario, Canada. Restoration Ecology, 19, 70–77.

Zeigler, S., Neel, M., Oliveira, L., Raboy, B. & Fagan, W.F. (2011) Conspecific and heterospecific attraction in assessments of functional connectivity. Biodiversity and Conservation, 20, 2779–2796.

Zeng, Z. & Brown, J.H. (1987) Population ecology of a desert rodent: Dipodomys merriami in the Chihuahuan Desert. Ecology, 68, 1328–1340.

Zenger, K.R., Eldridge, M.D.B. & Cooper, D.W. (2003) Intraspecific variation, sex-biased dispersal and phylogeography of the eastern grey kangaroo (Macropus giganteus). Heredity, 91, 153–160.

Zhan, X.J., Zhang, Z.J., Wu, H., Goossens, B., Li, M., Jiang, S.W., Bruford, M.W. & Wei, F.W. (2007) Molecular analysis of dispersal in giant pandas. Molecular Ecology, 16, 3792–3800.

Zimen, E. (1984) Long-range movements of the red fox (Vulpes vulpes). Acta Zoologica Fennica, 171, 267–270.

Zimmermann, F., Breitenmoser-Wursten, C., et al. (2005) Natal dispersal of Eurasian lynx (Lynx lynx) in Switzerland. Journal of Zoology, 267, 381–388.

**Swimming fish:**

Altena, E.R. (2003) Smallmouth bass movement and habitat use in the upper Mississippi River, St. Cloud to Coon Rapids. Montrose.

Anderson, J.T., Nuttle, T., Saldana Rojas, J.S., Pendergast, T.H. & Flecker, A.S. (2011) Extremely long-distance seed dispersal by an overfished Amazonian frugivore. *Proceedings of the Royal Society B: Biological Sciences*, 278, 3329–3335.

Barth, C.C., Anderson, W.G., Henderson, L.M. & Peake, S.J. (2011) Home range size and seasonal movement of juvenile lake sturgeon in a large river in the Hudson Bay drainage basin. Transactions of the American Fisheries Society, 140, 1629–1641.

Berland, G., Nickelsen, T., Heggenes, J., Økland, F., Thorstad, E.B. & Halleraker, J. (2004) Movements of wild Atlantic salmon parr in relation to peaking flows below a hydropower station. River Research and Applications, 20, 957–966.

Berra, T.M. & Gunning, G.E. (1972) Seasonal movement and home range of the longear sunfish, Lepomis megalotis (Rafinesque) in Louisiana. The American Midland Naturalist, 88, 368–375.

Bjornn, T.C. & Mallet, J. (1964) Movements of planted and wild trout in an Idaho river system. Transactions of the American Fisheries Society, 93, 70–76.

Bowerman, T. (2013) A multi-scale investigation of factors limiting bull trout viability. PhD Thesis, Utah State University, Logan, Utah.

Bozeman, E.L., Helfman, G.S. & Richardson, T. (1985) Population size and home range of American eels in a Georgia tidal creek. Transactions of the American Fisheries Society, 114, 821–825.

Bramblett, R.G. (1996) Habitats and movements of pallid and shovelnose sturgeon in the Yellowstone and Missouri rivers, Montana and North Dakota. PhD Thesis, Montana State University, Bozeman, Montana.

Bridcut, E.E. & Giller, P.S. (1993) Movement and site fidelity in young brown trout Salmo trutta populations in a southern Irish stream. Journal of Fish Biology, 43, 889–899.

Brown Jr, E.H. (1961) Movement of native and hatchery-reared game fish in a warm-water stream. Transactions of the American Fisheries Society, 90, 449–456.

Bryant, M.D., Lukey, M.D., McDonell, J.P., Gubernick, R.A. & Aho, R.S. (2009) Seasonal movement of Dolly Varden and cutthroat trout with respect to stream discharge in a second-order stream in Southeast Alaska. North American Journal of Fisheries Management, 29, 1728–1742.

Clapp, D.F., Clark, R.D. & Diana, J.S. (1990) Range, activity, and habitat of large, free-ranging brown trout in a Michigan Stream. Transactions of the American Fisheries Society, 119, 1022–1034.

Connor, W., Steinhorst, R. & Burge, H. (2003) Migrational behavior and seaward movement of wild subyearling fall chinook salmon in the Snake River. North American Journal of Fisheries Management, 23, 414–430.

Crook, D.A. (2004) Is the home range concept compatible with two species of lowland river fish? Journal of Animal Ecology, 73, 353–366.

Donnelly, R.E., Caffrey, J.M. & Tierney, D.M. (1998) Movements of a bream (Abramis brama (L.)), rudd x bream hybrid, tench (Tinca tinca (L.)) and pike (Esox lucius (L.)) in an Irish canal habitat. Hydrobiologia, 371/372, 305–308.

Ebner, B.C. & Thiem, J.D. (2009) Monitoring by telemetry reveals differences in movement and survival following hatchery or wild rearing of an endangered fish. Marine and Freshwater Research, 60, 45–57.

Fajan, O.F. (1962) The influence of stream stability on homing behavior of two smallmouth bass populations. Transactions of the American Fisheries Society, 91, 346–349.

Fickling, N.J. & Lee, R.L.G. (1985) A study of the movements of the zander, Lucioperca lucioperca L., population of two lowland fisheries. Aquaculture Research, 16, 377–393.

Freeman, M.C. (1995) Movements by two small fishes in a large stream. Copeia, 1995, 361–367.

Geeraerts, C., Ovidio, M., Verbiest, H., Buysse, D., Coeck, J., Belpaire, C. & Philippart, J.C. (2007) Mobility of individual roach Rutilus rutilus (L.) in three weir-fragmented Belgian rivers. Hydrobiologia, 582, 143–153.

Godinho, A.L., Kynard, B. & Godinho, H.P. (2007) Migration and spawning of female surubim (Pseudoplatystoma corruscans, Pimelodidae) in the São Francisco river, Brazil. Environmental Biology of Fishes, 80, 421–433.

Gresswell, R.E. & Hendricks, S.R. (2007) Population-scale movement of coastal cutthroat trout in a naturally isolated stream network. Transactions of the American Fisheries Society, 136, 238–253.

Hafs, A.W., Gagen, C.J. & Whalen, J.K. (2010) Smallmouth bass summer habitat use, movement, and survival in response to low flow in the Illinois Bayou, Arkansas. North American Journal of Fisheries Management, 30, 604–612.

Harcup, M.F., Williams, R. & Ellis, D. (1984) Movements of brown trout, Salmo trutta L., in the river Gwyddon, South Wales. Journal of Fish Biology, 24, 415–426.

Heggenes, J., Qvenild, T., Stamford, M.D. & Taylor, E.B. (2006) Genetic structure in relation to movements in wild European grayling (Thymallus thymallus) in three Norwegian rivers. Canadian Journal of Fisheries and Aquatic Sciences, 63, 1309–1319.

Hesthagen, T. (1988) Movements of brown trout, Salmo trutta, and juvenile Atlantic salmon, Salmo salar, in a coastal stream in northern Norway. Journal of Fish Biology, 32, 639–653.

Hilderbrand, R.H. & Kershner, J.L. (2000) Movement patterns of stream-resident cutthroat trout in Beaver Creek, Idaho – Utah. Transactions of the American Fisheries Society, 129, 1160–1170.

Huddleston, M. (2006) Dispersal, persistence, and areas of core use of re-introduced juvenile lake sturgeon in the Upper Tennessee River System. Master's Thesis, University of Tennessee, Knoxville.

Hurley, S.T., Hubert, W.A. & Nickum, J.G. (1987) Habitats and movements of shovelnose sturgeons in the upper Mississippi River. Transactions of the American Fisheries Society, 116, 655–662.

Jellyman, D.J. & Sykes, J.R.E. (2003) Diel and seasonal movements of radio-tagged freshwater eels, Anguilla spp., in two New Zealand streams. Environmental Biology of Fishes, 66, 143–154.

Jenkins, D.G., Brescacin, C.R., Duxbury, C.V., Elliott, J.A., Evans, J.A., Grablow, K.R., et al. (2007) Does size matter for dispersal distance? Global Ecology and Biogeography, 16, 415–425.

Jones, M.J. & Stuart, I.G. (2007) Movements and habitat use of common carp (Cyprinus carpio) and Murray cod (Maccullochella peelii peelii) juveniles in a large lowland Australian river. Ecology of Freshwater Fish, 16, 210–220.

Kahler, T., Roni, P. & Quinn, T. (2001) Summer movement and growth of juvenile anadromous salmonids in small western Washington streams. Canadian Journal of Fisheries and Aquatic Sciences, 58, 1947–1956.

Kanno, Y., Letcher, B.H., Coombs, J.A., Nislow, K.H. & Whiteley, A.R. (2014) Linking movement and reproductive history of brook trout to assess habitat connectivity in a heterogeneous stream network. Freshwater Biology, 59, 142–154.

Kanno, Y., Vokoun, J.C. & Letcher, B.H. (2011) Fine-scale population structure and riverscape genetics of brook trout (Salvelinus fontinalis) distributed continuously along headwater channel networks. Molecular Ecology, 20, 3711–3729.

Kirk, J.P., Killgore, K.J., Morrow, J.V., Lamprecht, S.D. & Cooke, D.W. (2001) Movements of triploid grass carp in the Cooper River, South Carolina. Journal of Aquatic Plant Management, 39, 59–62.

Knouft, J.H. & Spotila, J.R. (2002) Assessment of movements of resident stream brown trout, Salmo trutta L., among contiguous sections of stream. Ecology of Freshwater Fish, 11, 85–92.

Koed, A., Balleby, K., Mejlhede, P. & Aarestrup, K. (2006) Annual movement of adult pike (Esox lucius L.) in a lowland river. Ecology of Freshwater Fish, 15, 191–199.

Kulíšková, P., Horký, P., Slavík, O. & Jones, J.I. (2009) Factors influencing movement behaviour and home range size in ide Leuciscus idus. Journal of Fish Biology, 74, 1269–1279.Lin, S., Iizuka, Y. & Tzeng, W. (2012) Migration behavior and habitat use by juvenile Japanese eels. Zoological Studies, 51, 442–452.

Lin, S., Iizuka, Y. and Tzeng, W. (2012) Migration behavior and habitat use by juvenile Japanese eels. Zoological Studies 51, 442–452.

Lyons, J. & Lucas, M.C. (2002) The combined use of acoustic tracking and echosounding to investigate the movement and distribution of common bream (Abramis brama) in the River Trent, England. Hydrobiologia, 483, 265–273.

Makrakis, M.C., Miranda, L.E., Makrakis, S., Fontes Júnior, H.M., Morlis, W.G., Dias, J.H.P. & Garcia, J.O. (2012) Diversity in migratory patterns among Neotropical fishes in a highly regulated river basin. Journal of Fish Biology, 81, 866–881.

Makrakis, M.C., Miranda, L.E., Makrakis, S., Fernandez, D.R., Garcia, J.O. and Dias, J.H.P. (2007) Movement patterns of armado, Pterodoras granulosus, in the Paraná River Basin. Ecology of Freshwater Fish 16, 410–416.

Martinelli, T.L. & Shively, R.S. (1997) Seasonal distribution, movements and habitat associations of northern squawfish in two lower Columbia River reservoirs. Regulated Rivers: Research & Management, 13, 543–556.

McGrath, P.E., Hilton, E.J. & Musick, J.A. (2012) Seasonal distributions and movements of longnose gar (Lepisosteus osseus) within the York River System, Virginia. Southeastern Naturalist, 11, 375–386.

McLellan, J., McLellan, H. & Scholz, A. (2002) Assessment of the Lake Roosevelt walleye population: compilation of 1997-1999 data. 1999-2000 Annual Report, Project No 199404300, Portland.

Moen, C. & Scarnecchia, D. (1992) Paddlefish movements and habitat use in Pool 13 of the Upper Mississippi River during abnormally low river stages and discharges. North American Journal of Fisheries Management, 12, 744–751.

Mollenhauer, R., Wagner, T., Kepler, M. V. & Sweka, J.A. (2013) Fall and early winter movement and habitat use of wild brook trout. Transactions of the American Fisheries Society, 142, 1167 – 1178.

Muhlfeld, C.C. & Marotz, B. (2005) Seasonal movement and habitat use by subadult bull trout in the upper Flathead River system, Montana. North American Journal of Fisheries Management, 25, 797–810.

Naesje, T. (2008) Dispersal in stream-living brown trout (Salmo trutta). Master Thesis, University of Oslo, Oslo.

Nichols, P.R. & Miller, R.V. (1967) Seasonal movements of striped bass, Roccus saxatilis (Walbaum), tagged and released in the Potomac River, Maryland, 1959-1961. Chesapeake Science, 8, 102–124.

Økland, F., Thorstad, E.B., Hay, C.J., Næsje, T.F. & Chanda, B. (2005) Patterns of movement and habitat use by tigerfish (Hydrocynus vittatus) in the upper Zambezi River (Namibia). Ecology of Freshwater Fish, 14, 79–86.

Osborne, M.W., Ling, N., Hicks, B.J. & Tempero, G.W. (2009) Movement, social cohesion and site fidelity in adult koi carp, Cyprinus carpio. Fisheries Management and Ecology, 16, 169–176.

Paller, M.H., Fletcher, D.E., Jones, T., Dyer, S.A., Isely, J.J. & Littrell, J.W. (2005) Potential of largemouth bass as vectors of 137Cs dispersal. Journal of Environmental Radioactivity, 80, 27–43.

Paragamian, V.L. & Wakkinen, V.D. (2008) Seasonal movement of burbot in relation to temperature and discharge in the Kootenai River, Idaho, USA and British Columbia. American Fisheries Society Symposium, 59, 55–77.

Peňáz, M., Baruš, V., Prokeš, M. & Homolka, M. (2002) Movements of barbel, Barbus barbus (pisces: Cyprinidae). Folia Zoologica, 51, 55–66.

Pesoa, N. A & Schulz, U.H. (2010) Diel and seasonal movements of grumatã Prochilodus lineatus (Valenciennes 1836) (Characiformes: Prochilodontidae) in the Sinos River, Southern Brazil. Brazilian journal of biology, 70, 1169–77.

Peters, L.M., Pegg, M.A. & Reinhardt, U.G. (2006) Movements of adult radio-tagged bighead carp in the Illinois River. Transactions of the American Fisheries Society, 135, 1205–1212.

Peterson, D. & Fausch, K. (2003) Upstream movement by nonnative brook trout (Salvelinus fontinalis) promotes invasion of native cutthroat trout (Oncorhynchus clarki) habitat. Canadian Journal of Fisheries and Aquatic Sciences, 60, 1502–1516.

Pettit, S.W. & Wallace, R.L. (1975) Age, growth, and movement of mountain whitefish, Prosopium williamsoni (Girard), in the North Fork Clearwater River, Idaho. Transactions of the American Fisheries Society, 1, 68–76.

Petty, J.T., Hansbarger, J.L., Huntsman, B.M. & Mazik, P.M. (2012) Brook trout movement in response to temperature, flow, and thermal refugia within a complex Appalachian riverscape. Transactions of the American Fisheries Society, 141, 1060–1073.

Popoff, N.D. & Neumann, R.M. (2005) Range and movement of resident holdover and hatchery brown trout tagged with radio transmitters in the Farmington River, Connecticut. North American Journal of Fisheries Management, 25, 413–422.

Roghair, C. & Dolloff, C. (2005) Brook trout movement during and after recolonization of a naturally defaunated stream reach. North American Journal of Fisheries Management, 25, 777–784.

Schultz, U.H. & Leal, M.E. (2012) Effects of stocking density on dispersal behavior of Brazilian freshwater dourado (Salminus brasiliensis) in a subtropical river headwater. Neotropical Ichthyology, 10, 409–415.

Simpson, R.R. & Mapleston, A.J. (2002) Movements and habitat use by the endangered Australian freshwater Mary River cod, Maccullochella peelii mariensis. Environmental Biology of Fishes, 65, 401–410.

Skalski, G. & Gilliam, J.F. (2000) Modeling diffusive spread in a heterogeneous population: a movement study with stream fish. Ecology, 81, 1685–1700.

Skyfield, J.P. & Grossman, G.D. (2008) Microhabitat use, movements and abundance of gilt darters (Percina evides) in southern Appalachian (USA) streams. Ecology of Freshwater Fish, 17, 219–230.

Slavík, O., Bartoš, L. & Mattas, D. (2005) Does stream morphology predict the home range size in burbot? Environmental Biology of Fishes, 74, 89–98.

Smithson, E.B. & Johnston, C.E. (1999) Movement pattern of stream fishes in a Ouachita highlands stream: an examination of the restricted movement paradigm. Transactions of the American Fisheries Society, 128, 847–853.

Solomon, D.J. (1976) Movements of brown trout Salmo trutta L. in a chalk stream. Journal of Fish Biology, 9, 411–423.

Starcevich, S.J. (2005) Seasonal movement patterns and habitat use of westernslope cutthroat trout in two headwater tributary streams of the John Day River. Master Thesis, Oregon State University, Portland.

Steingrímsson, S.Ó. & Grant, J.W.A. (2003) Patterns and correlates of movement and site fidelity in individually tagged young-of-the-year Atlantic salmon (Salmo salar). Canadian Journal of Fisheries and Aquatic Sciences, 60, 193–202.

Stickler, M., Enders, E.C., Pennell, C.J., Cote, D., Alfredsen, K. & Scruton, D. A. (2008) Stream gradient-related movement and growth of Atlantic salmon parr during winter. Transactions of the American Fisheries Society, 137, 371–385.

Stott, B. (1967) The movements and population densities of roach (Rutilus rutilus (L.)) and gudgeon (Gobio gobio (L.)) in the River Mole. Journal of Animal Ecology, 36, 407–423.

Thorstad, E.B., Hay, C.J., Naesje, T.F. & Okland, F. (2001) Movements and habitat utilization of three cichlid species in the Zambezi River, Namibia. Ecology of Freshwater Fish, 10, 238–246.

Travnichek, V.H. (2004) Movement of flathead catfish in the Missouri River: examining opportunities for managing river segments for different fishery goals. Fisheries Management and Ecology, 11, 89–96.

Vilizzi, L., Copp, G.H., Carter, M.G. & Peňáz, M. (2006) Movement and abundance of barbel, Barbus barbus, in a mesotrophic chalk stream in England. Folia Zoologica, 55, 183–197.

Westhoff, J.T., Paukert, C., Ettinger-Dietzel, S., Dodd, H. & Siepker, M. (2016) Behavioural thermoregulation and bioenergetics of riverine smallmouth bass associated with ambient cold-period thermal refuge. Ecology of Freshwater Fish, 25, 72–85.

Winter, H.V. & Fredrich, F. (2003) Migratory behaviour of ide: a comparison between the lowland rivers Elbe, Germany, and Vecht, The Netherlands. Journal of Fish Biology, 63, 871–880.

Wooley, C.M. & Crateau, E.J. (1983) Biology, population estimates, and movement of native and introduced striped bass, Apalachicola River, Florida. North American Journal of Fisheries Management, 3, 383–394.

Young, M.K. (2011) Generation-scale movement patterns of cutthroat trout (Oncorhynchus clarkii pleuriticus) in a stream network. Canadian Journal of Fisheries and Aquatic Sciences, 68, 941–951.
